# Supplementation of L-aspartate corrects MASLD and MASH in mice by inhibiting platelet–hepatocyte interaction-mediated mitochondrial fragmentation via the ATP–P2X7–NEK7–DRP1 axis

**DOI:** 10.1038/s12276-026-01648-9

**Published:** 2026-02-13

**Authors:** Wen-Jie Cao, Rui Su, Hui-Ling Fu, Jun-Jie Wu, Lin-sheng Huang, Fei-fei Liu, Jin Liu, Zhong-Ping Jiang, Cong-Jun Xu, Yong Rao, Ling Huang

**Affiliations:** 1 Key Laboratory of Tropical Biological Resources of Ministry of Education, School of Pharmaceutical Sciences, Hainan University, Haikou, China; 2 Department of Hepatopancreatobiliary Surgery, Taihe Hospital, Shiyan, China

**Keywords:** Calcium and phosphate metabolic disorders, Drug development

## Abstract

Metabolic dysfunction-associated steatotic liver disease (MASLD) is a worldwide prevalent metabolic disorder with increasing demands for therapeutic agents. l-aspartate is a nonessential amino acid that has great potential for curing liver disease. However, the therapeutic potential of l-aspartate against MASLD and its severe form metabolic dysfunction-associated steatohepatitis (MASH), as well as its metabolic regulation mode, are not well documented. Here we found that plasma and liver l-aspartate levels were decreased and negatively correlated with the severity of MASLD in mice and humans. l-aspartate supplementation in mice reversed the manifestations of both MASLD and MASH and these were correlated with improvements in hepatic mitochondrial quality and oxidation. The results of joint transcriptome and metabolomics analyses revealed that the metabolite cGMP and platelet activation were highly annotated after a single l-aspartate treatment. Notably, l-aspartate treatment increased cGMP levels in platelets and blocked platelet activation and aggregation, thereby suppressing activated platelet-derived ATP secretion and its mediated P2X7–NEK7–DRP1 axis hyperactivation in hepatocytes. Correspondingly, l-aspartate addition reversed the ATP-induced increases in oleatic acid-induced mitochondrial fragmentation and lipid accumulation. Interestingly, treatment with either the antiplatelet agent aspirin or the P2X7 inhibitor or NEK7 knockdown corrected oleatic acid + ATP-induced exacerbations of mitochondrial fragmentation and lipid accumulation in hepatocytes or ameliorated MASLD in mice. Notably, the l-aspartate increased cGMP levels in platelets was correlated with reductions in the plasma level of its inducers, including ADP and thrombin. These data together indicate that activated platelet-mediated mitochondrial fragmentation in hepatocytes is a pivotal driving force for MASLD and MASH. Blocking platelet activation underlies the therapeutic potential and metabolic regulation of l-aspartate against MASLD and MASH.

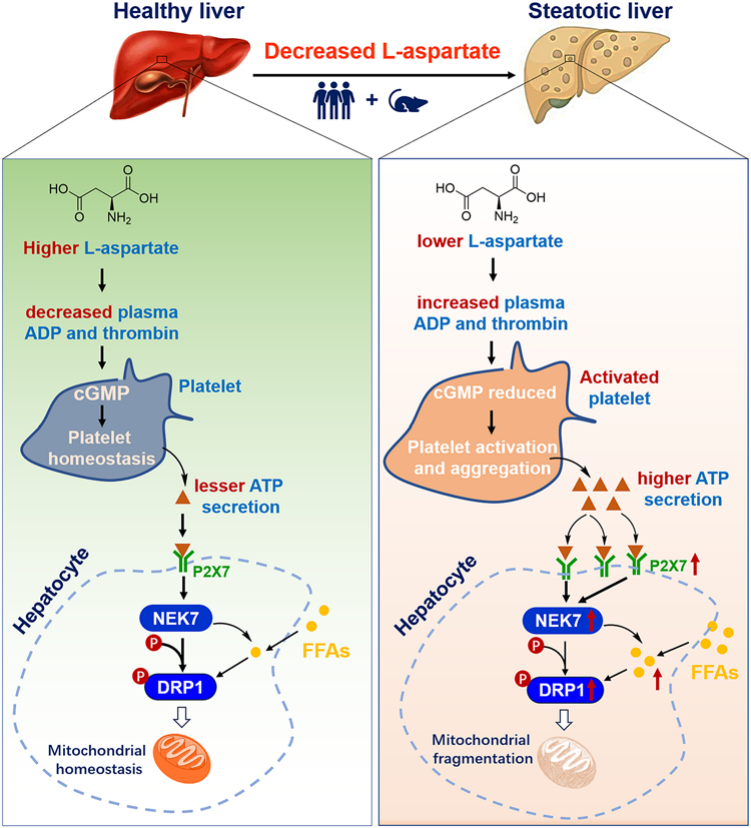

## Highlights

- Hepatic l-aspartate levels are decreased in mice and humans with MASLD.
- Treating with l-aspartate efficiently reverses MASLD and MASH, alongside improvements in hepatic mitochondrial quality.
- l-aspartate inhibits platelet activation and its mediated release of the toxic substance ATP.
- ATP induces P2X7–NEK7–DRP1 axis hyperactivation in hepatocytes, contributing to mitochondrial fragmentation and lipid accumulation.
- l-aspartate is a promising anti-MASLD candidate that inhibits ATP-mediated platelet and hepatocyte interactions.

## l-aspartate reverses liver disease by targeting platelet activation

Metabolic dysfunction-associated steatotic liver disease (MASLD) is a common liver condition linked to obesity and diabetes. Currently, only one drug, resmetirom, is approved for treatment, but it has limited effectiveness. Researchers are exploring new treatments. Here researchers aimed to investigate the potential of l-aspartate, a nonessential amino acid, in treating MASLD. The study involved experiments on mice and analysis of human liver samples. The researchers found that l-aspartate levels were lower in the livers of both mice and humans with MASLD. They treated mice with l-aspartate and observed improvements in liver health and mitochondrial function. The study also showed that l-aspartate reduced platelet activation, which helped prevent liver damage. The results suggest that l-aspartate could be a promising treatment for MASLD by improving mitochondrial health and reducing harmful platelet activity.

This summary was initially drafted using artificial intelligence, then revised and fact-checked by the author.

## Introduction

Metabolic dysfunction-associated steatotic liver disease (MASLD), also known as nonalcoholic fatty liver disease, includes a series of liver pathological phenotypes manifested by heavy lipid accumulation^1^. MASLD has rapidly become the most prevalent chronic liver disease and is predicted to affect up to 38% of the global population^2^; its incidence is estimated to increase exponentially since the increasing epidemics of obesity and diabetes^3^. MASLD can progressively deteriorate into metabolic dysfunction-associated steatohepatitis (MASH), hepatic fibrosis and even hepatocellular carcinoma. So far, only the thyroid hormone receptor-beta (THR-β) agonist resmetirom has been approved to treat MASLD and MASH, with ~26–30% clinical efficacy^4^. The identification of novel therapeutic agents against MASLD and MASH is highly important in the field of medicine and remains still challenging.

The pathogenesis of MASLD involves multiple biological processes, including insulin resistance, mitochondrial dysfunction, lipid metabolism dysfunction, lipotoxicity, oxidative stress and inflammation^5,6^. These processes communicate individually or together to exacerbate MASLD. Mitochondria are the main organelles that oxidize lipids and glucose, and high nutrient intake in hepatocytes impedes mitochondrial oxidation and induces mitochondrial damage, triggering the accumulation of toxic substances, including reactive oxygen species (ROS), cytochrome *C* and fragmented mitochondrial DNA (mtDNA), which not only impedes redox homeostasis in the liver but also induces lipid metabolism-related enzyme dysfunction, thereby exacerbating liver injury^7^. In addition, excessive lipid overload inhibits mitophagy, leading to the aggressive accumulation of damaged mitochondria and exacerbating lipid accumulation and liver injury. Hence, maintaining mitochondrial health or remodeling mitochondrial homeostasis is considered one of the most practicable therapeutic strategies against MASLD.

l-aspartate, a nonessential amino acid involved in the tricarboxylic acid cycle and nitrogen metabolism, is clinically used as a protective agent for acute liver injury and for the treatment of hepatic encephalopathy^8,9^. Recently, our studies revealed that increasing l-aspartate levels in the liver through pharmacological interventions (for example, gut microbes or dietary supplementation with l-aspartate) improved hepatic mitochondrial oxidation, resulting in liver injury or liver fibrosis after long-term drug exposure^10^. Given its mode of action, l-aspartate is theoretically considered a potential therapeutic agent for MASLD and MASH. However, the therapeutic potential of l-aspartate against MASLD/MASH and the key mechanisms underlying its metabolic regulation in the liver remain elusive.

In the present study, we first aimed to determine the correlations between liver l-aspartate levels and indicators of MASLD in both mice and humans. Second, we explored the therapeutic potential of l-aspartate in treating MASLD and MASH in relevant mouse models. Third, we revealed the crucial mechanism underlying hepatic mitochondrial quality regulation. This process was carried out by performing joint transcriptome and metabolomics analyses of metabolites and their associated pathological pathways correlated with mitochondrial dysfunction. Our data demonstrated that l-aspartate levels are decreased in the livers of mice with MASLD and humans. Instead, l-aspartate treatment in mice ameliorated both manifestations of MASLD and MASH alongside improvements in mitochondrial quality and oxidation in the liver. These effects were correlated with increased cGMP levels in platelets, leading to inhibition of platelet activation (induced by high-fat and high-cholesterol (HFC) diet feeding) in the liver as well as its derived ATP release, thereby suppressing ATP-induced lipid accumulation and mitochondrial fragmentation in hepatocytes *via* suppression of the P2X7–NEK7–DRP1 axis.

## Materials and methods

### Animal study

All animal care and experimental procedures were carried out in accordance with the Guide for the Care and Use of Laboratory Animals of the National Institutes of Health. The protocols were approved by the Hainan University Committee on Animal Ethics for the Use of Laboratory Animals and were conducted in accordance with the Animal Welfare Legislation of China (approval no. HPIACUC2023074). Every effort was made to minimize the use of the animals and their discomfort. Eight-week-old male C57BL/6J mice were fed with regular chow (RC) or HFC (60% fat and 1% cholesterol; Research Diet, M21051101) for 0, 2, 5, 8, 15 or 24 weeks, then livers and plasma were collected for the determination of l-aspartate level and indicators of MASLD.

The anti-MASLD efficacies of l-aspartate were evaluated in HFC diet-fed MASLD mice after 12 weeks feeding. Mice were then randomly separated into three subgroups to receive saline, l-aspartate (100 mg/kg, calculated according to its clinical dosage)^10^ or CL316243 (0.5 mg/kg) every other day for 76 days. The anti-MASH efficacies of l-aspartate were evaluated in methionine- and choline-deficient (MCD) diet (Research Diets, A02082002BR)-fed MASH mice after 4 weeks feeding. l-aspartate (100 mg/kg) or ocaliva (20 mg/kg) was administrated every 2 days for another 6 weeks.

### Human liver sample collection

Human liver tissues obtained were from 14 healthy populations and 26 MASLD populations, with plasma chemicals, liver pathology and ultrasonic examinations. The patients underwent open abdominal surgery (sleeve gastrectomy, elective cholecystectomy, Roux en Y bypass or explorative laparotomy) in accordance with The Code of Ethics of the World Medical Association (Declaration of Helsinki). The protocols have been approved by the ethics committee of the TaiHe hospital, Shiyan, Hubei, China (no. WZ2025005). The participants gave written informed consent before taking part in the study.

### Liver pathological examinations

The liver slides were subjected to hematoxylin–eosin (H&E) and oil red O staining to assess liver steatosis and hepatocyte ballooning. The NAS activity score was evaluated as previously described^11^.

### Hepatic l-aspartate levels and gene expression quantification

The level of plasma or hepatic l-aspartate was measured using the l-aspartate ELISA kit, as previously reported^12^. Briefly, 10 μl homogenized liver lysate and plasma or standards were added to the pretreated 96-well plates, mixed with 40 μl dilution buffer and then incubated with the 50 μl HRP-labeled competitive agents at 37 °C for 1 h. After washing, the substrates were added and incubated for 15 min at dark, then the reactions were terminated by adding the 50 μl stopping buffer. The absorbance was determined at 450 nm and the l-aspartate level was calculated by normalizing the protein content. Total RNA was isolated and target genes expression were determined using the 2^−ΔΔ*Ct*^ method as previously reported^12^.

### Immunoblot and immunofluorescence analysis

Immunoblotting and immunofluorescence assays were performed as previously reported^13^. Cells or tissues were lysed and homogenized, proteins were isolated and subjected to SDS–PAGE and then blocked for 30 min. Membranes were incubated with antibodies and protein bands were visualized. The antibodies used are listed in Supplementary Table 1. For immunofluorescence, the fixed cells or liver sections were incubated with CD68, Collagen I, TFAM, PGC-1α or CD42b and NEK7 at 4 °C overnight and then costained with a fluorescently labeled secondary antibody at 37 °C for 1 h. The images were captured.

### FFA uptake analysis

To measure free fatty acid (FFA) uptake, hepatocytes were treated with oleatic acid (OA) or OA + l-aspartate for 24 h and then the medium was replaced with fresh culture medium containing the TF2-C12 FA probe (Abcam, ab176768) at 0.5 μΜ. The fluorescence was measured at an excitation wavelength of 485 nm and an emission wavelength of 515 nm every 30 s for 20 min as previously reported^14^.

### Whole-body metabolic determination

Oxygen consumption and energy expenditure were determined using the CLAMS system (Promethion, Sable Systems International). After 48 h acclimation, VO_2_, VCO_2_ and energy expenditure were measured over the next 72 h. Voluntary activity was monitored every 15 min. Heat production and the respiratory exchange ratio (RER) were calculated^14^.

### Primary mouse hepatocytes isolation and OA induction

Primary mouse hepatocytes were isolated from 6-week-old male C57BL/6J mice as previously reported^15^. For induction, primary mouse hepatocytes were treated with 0.75 mM OA (Sigma, O1008) for 24 h.

### Platelet isolation and induction

Platelets from plasma or livers were isolated using the methods as previously described^16^. Whole blood obtained from mice via cardiac puncture was drawn into a vacutainer containing 3.8% sodium citrate (1/9, v/v). Anticoagulated blood was further centrifuged to prepare the platelet-rich plasma. Platelet-rich plasma was centrifuged and the isolations were then resuspended in Tyrode’s buffer and the suspended platelet density was adjusted to 10^8^ cells/ml. For the isolation of platelets from liver, the liver tissue was cut into small pieces and digested with 0.05% of collagenase Type IV at 37 °C for 20 min, then centrifuged at 1,000 rpm/min at room temperature for 5 min and the precipitate was isolated and washed twice. Then, the precipitate resuspended in Tyrode’s buffer containing 0.5 µg CD61 antibody for isolation through flow cytometry. Before the formal experiment, platelets were left to stand at room temperature for about 30 min. Washed platelets containing 1 mM CaCl_2_ were stimulated with thrombin (0.1 U/ml) or ADP (20 μM) at 37 °C for 5 min to activate them.

### Mitochondrial morphology analysis

Hepatocytes were stained with the 0.5 µM Mito-Tracker (Thermo Scientific, M22426) in the dark for 10 min. Mitochondrial morphology was captured using the Olympus FV3000 software equipped with a 63×/1.4 numerical aperture objective. The ImageJ software with MiNA (specific for mitochondrial morphology analysis) was used to quantify the mitochondrial length and the number of tubular, fragmented and mixed mitochondria in hepatocytes, as reported previously^17,18^. Briefly, the captured pictures were loaded to the ImageJ software, processed by unsharp mask and enhance local contrast analysis, then the filter was used at ‘median’ to reduce noise. After that, the images were converted to binary by thresholding, where a foreground pixel is assigned the maximum value 255 and background pixels are assigned the minimum possible value 0. Finally, the binary image was then converted to a skeleton using the ‘Skeletonize’ feature. All pixels within a skeleton were then grouped into three categories: a single pixel (fragmented), unbranched structures with two or more pixels (mixed) and networks of mitochondrial structures with at last a single node and three branches (tubular). All analyses performed by the software were manually confirmed. Data are the mean values from ~3– 5 images and 100 cells. The same cells were used to measure mitochondrial morphology.

### Statistical analysis

Data were obtained from four independent experiments or ten mice in each group. Data are expressed as mean ± s.e.m. Differences between two groups were analyzed by a Student’s *t*-test using GraphPad Prism. A *P* value ≤0.05 was considered statistically significant.

## Results

### Hepatic l-aspartate levels negatively correlate with MASLD in mice and humans

**Fig. 1:**
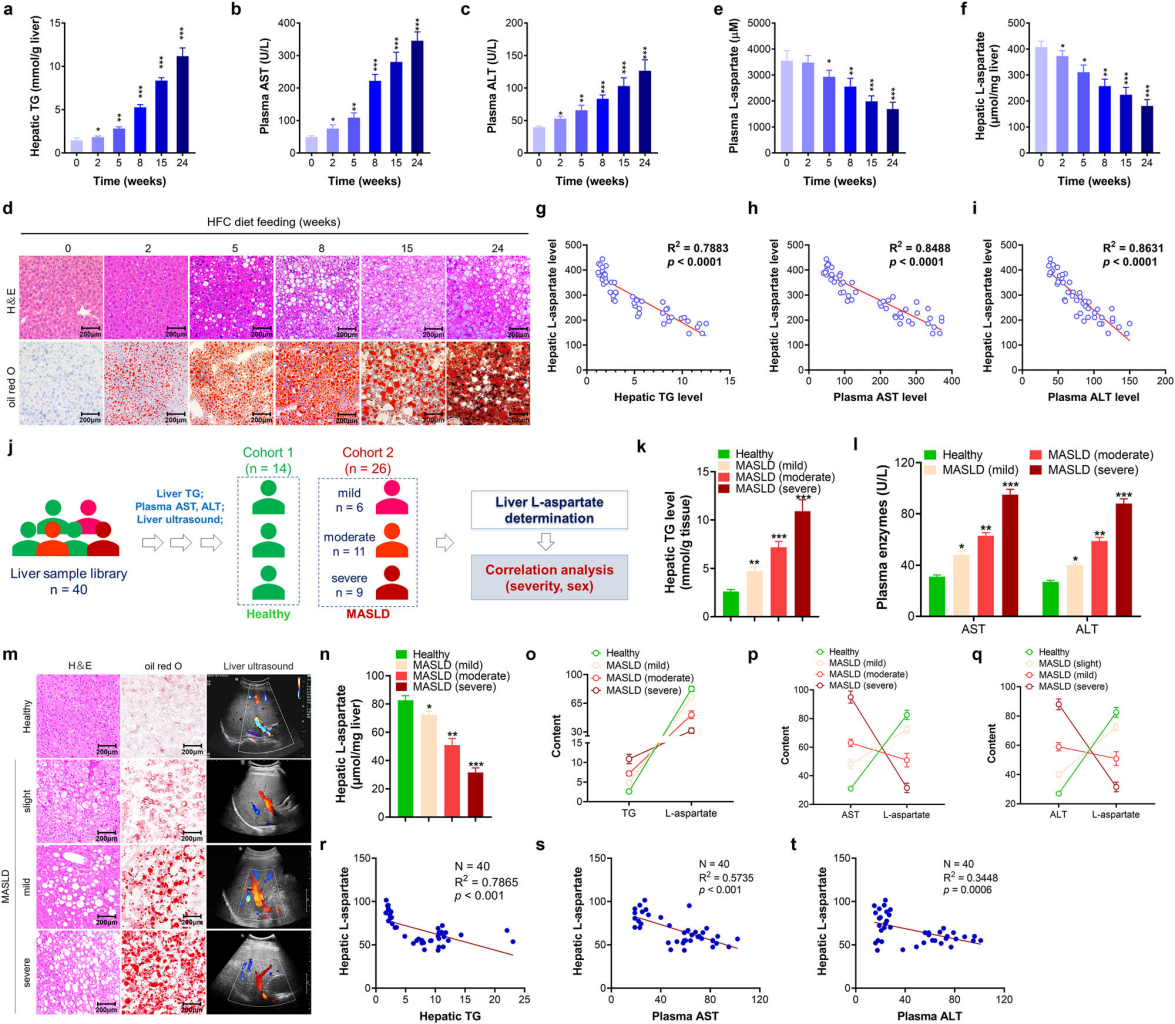
Hepatic l-aspartate level decreases in mice and humans with MASLD. **a**–**i** In 8-week-old male C57BL/6J mice fed with an HFC diet for 0, 2, 5, 8, 15 or 24 weeks, the livers and plasma were isolated for the following examinations: hepatic TG levels (**a**), plasma AST (**b**) and ALT (**c**) levels, H&E and oil red O staining (**d**) (scale bar, 200 μm), plasma (**e**) and hepatic (**f**) l-aspartate levels, and the correlation between hepatic TG (**g**), plasma AST (**h**) or ALT (**i**) levels and hepatic l-aspartate content. *N* = 10 mice/group. *P < 0.05, **P < 0.01 and ***P < 0.001, versus the RC mice. **j** A schematic diagram of human liver sample characterization. **k** Quantification of hepatic TG levels. **l** The determination of plasma AST and ALT levels. **m** H&E, oil red O staining and liver ultrasound examinations. Scale bar, 200 μm. **n** Hepatic l-aspartate quantification. **o**–**q** Correlation analyses between hepatic TG (**o**), plasma AST (**p**) or ALT (**q**) levels and hepatic l-aspartate contents in humans with different stages of MASLD. **r**–**t** Correlation analysis between hepatic TG (**r**), plasma AST (**s**) or ALT (**t**) levels and hepatic l-aspartate content in human patients. *P < 0.05, **P < 0.01 and ***P < 0.001 versus the healthy population.

To explore the correlation between hepatic l-aspartate levels and the development of MASLD, we fed mice a HFC diet for 0, 2, 5, 8, 15 or 24 weeks. As expected, we found that mice fed with a HFC diet displayed typical manifestations of MASLD, including hepatic steatosis and liver injury, as indicated by increases in the hepatic triglycerides (TG) and plasma aspartate transaminase (AST) and alanine transaminase (ALT) levels (Fig. 1a–c). H&E and oil red O staining also confirmed these effects of MASLD in the liver (Fig. 1d). Alongside these exacerbations, plasma and hepatic l-aspartate levels gradually decreased (Fig. 1e,f). This reduction was not associated with the constituents of the diets used (Supplementary Table 2). Notably, the hepatic l-aspartate level was negatively correlated with the hepatic TG and plasma AST and ALT levels (Fig. 1g–i). These results were also found in the livers of MASH mice induced by HFC/MCD diet feeding^13^ (Supplementary Fig. 1). We also quantified hepatic l-aspartate levels in healthy and MASLD populations via a combination of hepatic TG, plasma AST and ALT level quantification and liver ultrasound analysis (Fig. 1j–m). Compared to healthy individuals, those with MASLD presented greater reductions in l-aspartate levels in the liver (Fig. 1n). Notably, hepatic l-aspartate levels gradually decreased alongside the deterioration of MASLD in humans, as indicated by increases in the levels of hepatic TG and plasma AST and ALT (Fig. 1o–t). Determination of l-aspartate levels in the livers of both the male and female patients with MASLD revealed decreases in the levels of hepatic l-aspartate but no difference was observed between the male and female populations (Supplementary Fig. 2), implying that sex differences only marginally affected the difference in l-aspartate levels in human patients with MASLD.

#### l-aspartate supplementation reverses MASLD and MASH in mice

**Fig. 2:**
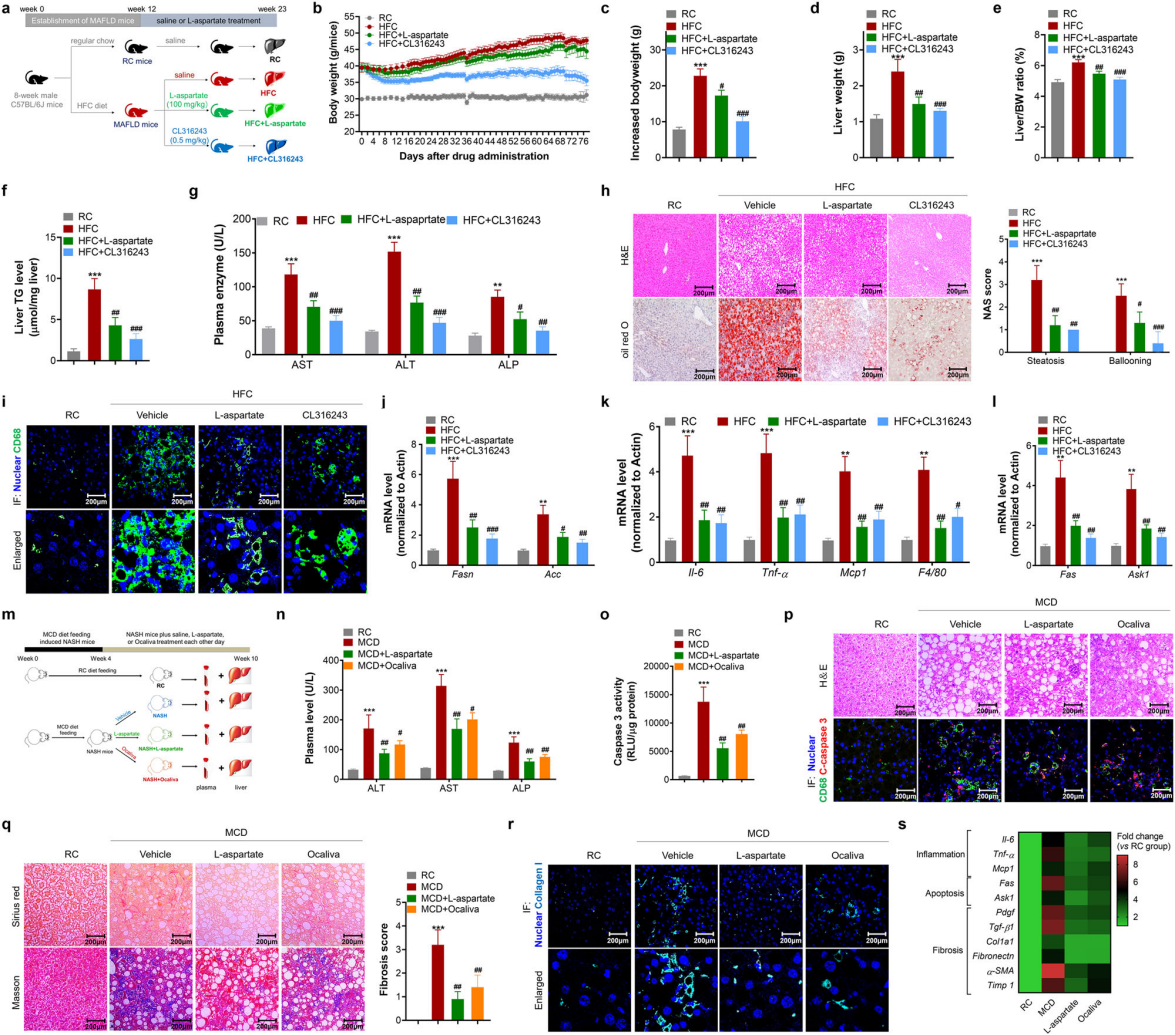
l-aspartate alleviates MASLD and MASH in mice. **a**–**l** Evaluation of the anti-MASLD action of l-aspartate in HFC diet-fed MASLD mice: a schematic diagram of l-aspartate treatment in MASLD mice (**a**); mouse bodyweight (BW) curve during l-aspartate treatment (**b**); determination of mouse bodyweight change (**c**); liver weight (**d**) and liver-to-BW (**e**) measurements; hepatic TG quantification (**f**); plasma AST, ALT and ALP determination (**g**); H&E and oil red O staining and NAS score evaluation (**h**) (scale bar, 200 μm); immunofluorescence analysis of CD68 (**i**) (scale bar, 200 μm); and determination of gene expression related to lipogenesis (**j**), inflammation (**k**) and apoptosis (**l**) in the liver. **m**–**s**, Evaluation of the anti-MASH action of l-aspartate in MCD diet-induced MASH mice: a schematic diagram of l-aspartate treatment in MASH mice (**m**); plasma AST, ALT and ALP determination (**n**); determination of caspase 3 activity (**o**); H&E staining and immunofluorescence analysis of CD68 and cleaved caspase 3 (**p**) (scale bar, 200 μm); Sirius red and masson staining (**q**) (scale bar, 200 μm); immunofluorescence analysis of collagen I (**r**) (scale bar, 200 μm); and determination of gene expression related to inflammation, apoptosis and fibrosis in the liver (**s**). *N* = 10 mice/group. *P < 0.05, **P < 0.01 and ***P < 0.001 versus the RC mice; ^#^*P* < 0.05, ^##^*P* < 0.01 and ^###^*P* < 0.001 versus the HFC or MCD control mice.

We then explored the therapeutic potential of l-aspartate in reversing MASLD and MASH in mice. After acclimation, mice were fed the HFC diet for 23 weeks, and l-aspartate (100 mg/kg, calculated according to its clinical dosage)^10^ was administered via intraperitoneal injection every other day for 11 weeks. The β3-AR agonist CL316243 was used as a positive control (Fig. 2a)^19^. Treatment with either CL316243 or l-aspartate efficiently decreased mouse bodyweight, liver weight and the ratio of liver weight to bodyweight (Fig. 2b–e). Analysis of hepatic TG and plasma liver enzymes revealed that either CL316243 or l-aspartate treatment decreased hepatic TG, plasma AST, ALT and alkaline phosphatase (ALP) levels (Fig. 2f–g). Moreover, H&E and oil red O staining further confirmed the therapeutic effects of l-aspartate and CL316243 against MASLD (Fig. 2h). Furthermore, we evaluated the anti-inflammatory effects of l-aspartate in the liver by examining the liver residue macrophage marker CD68. HFC diet feeding significantly increased CD68 signals in the liver, whereas a reversal effect was observed upon l-aspartate or CL316243 treatment (Fig. 2i). Consistently, gene expression analysis revealed that both CL316243 and l-aspartate treatment downregulated the levels of genes related to lipogenesis (*Fasn* and *Acc*), inflammation (*Il-6*, *Tnf-α*, *Mcp1* and *F4/80*) and apoptosis (*Fas* and *Ask1*) in the liver (Fig. 2j–l). Also, the preventative effects of l-aspartate were also observed in HFC diet-fed female mice after 8 weeks of treatment with l-aspartate (Supplementary Fig. 3). Notably, these therapeutic effects were not correlated with daily appetite inhibition (Supplementary Fig. 4a).

MASH is a severe stage of MASLD with fewer approved therapeutic agents. We also evaluated the anti-MASH efficacy of l-aspartate in MCD diet-fed mice, with the FXR agonist ocaliva selected as a positive control (Fig. 2m). As expected, MCD diet feeding induced severe hepatic injury, hepatocyte death, inflammation and histological fibrosis, whereas treatment with either l-aspartate or ocaliva restored all MASH phenotypes, including liver injury, inflammation and fibrosis (particularly histological fibrosis) (Fig. 2n–r). Similarly, gene expression analysis revealed that both l-aspartate treatment and ocaliva treatment downregulated the transcription levels of genes related to inflammation (*Il-6*, *Tnf-α* and *Mcp1*), apoptosis (*Fas* and *Ask1*) and fibrosis (*Pdgf*, *Tgf-β1*, *Col1a1*, *Fibronectin*, *α-SMA* and *Timp1*) in the liver (Fig. 2s).

#### l-aspartate improves mitochondrial quality in the livers of mice with MASLD

**Fig. 3:**
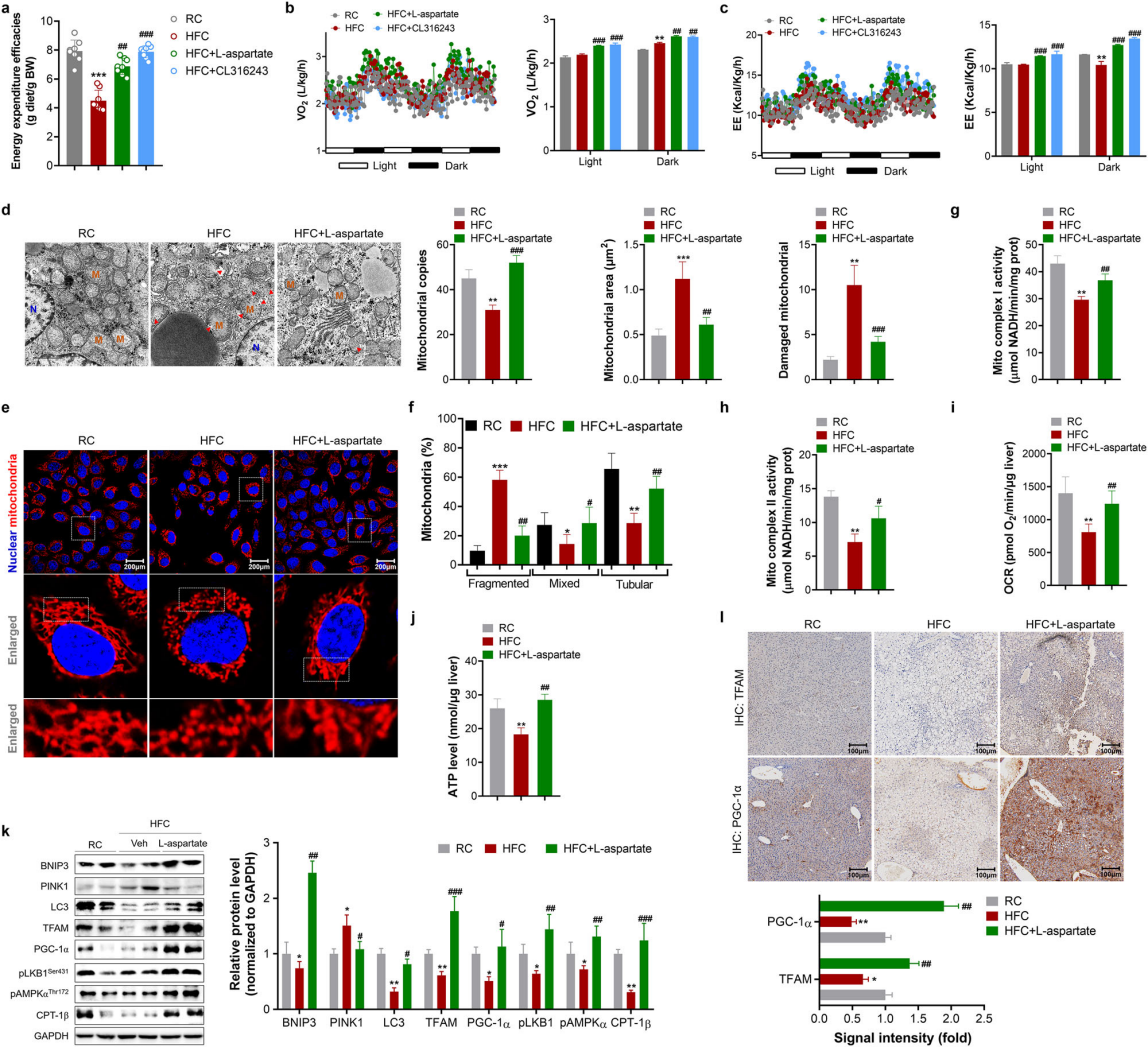
l-aspartate improves mitochondrial quality and oxidation in livers of HFC diet-induced MASLD mice. **a** Determination of the energy expenditure efficacy. **b**,**c** Determination of oxygen consumption (VO_2_; **b**) and energy expenditure (EE; **c**) using the CLAMPS system. **d** TEM analysis of mitochondria in liver and quantification. Scale bar, 2 µm. **e** Images of mitochondria in isolated primary mouse hepatocytes from the RC, HFC and HFC + l-aspartate mice. **f** Mitochondrial morphology quantification. **g**,**h** Measurements of mitochondrial complex I (**g**) and II (**h**) activities. **i** Evaluation of mitochondrial oxygen consumption rate (OCR) in the liver. **j** The determination of ATP production in isolated mitochondria. **k** Examination of protein levels related to mitophagy, mitobiogenesis and energy expenditure (left) and a densitometric analysis (right). **l** Immunohistochemistry analysis of TFAM and PGC-1α in liver. *N* = 10 mice/group. *P < 0.05, **P < 0.01 and ***P < 0.001 versus the RC mice; ^#^*P* < 0.05, ^##^*P* < 0.01 and ^###^*P* < 0.001 versus the HFC control mice.

Studies have emphasized that promoting energy expenditure is a practical and promising therapeutic strategy for combating metabolic disorders, including MASLD^20,21^. This finding has implications for identifying novel and drug-like molecules with high efficacy in facilitating energy expenditure in vivo. Next, we determined the metabolic improvement effects in mice. Measurement of energy expenditure efficacy in mice revealed that either CL316243 or l-aspartate treatment significantly reversed the decrease in energy expenditure efficacy in mice with MASLD (Fig. 3a). Whole-body metabolic assays in mice with MASLD revealed that treatment with either CL316243 or l-aspartate increased oxygen consumption, carbon dioxide production, and energy expenditure (Fig. 3b,c and Supplementary Fig. 4b). Notably, l-aspartate treatment increased physical activity but did not increase heat production, which distinguished from the β3-AR agonist CL316243 (Supplementary Fig. 4c–e).

Consistently, l-aspartate treatment corrected mitochondrial dysfunction in the liver. Transmission electron microscopy (TEM) analysis revealed that HFC diet feeding decreased the total number of mitochondria, increased the size of the mitochondria and increased the number of damaged mitochondria (Fig. 3d). Imaging mitochondria in primary mouse hepatocytes isolated from the livers of RC or HFC diet-fed or l-aspartate-treated HFC diet-fed mice demonstrated that HFC diet feeding reduced the number of mitochondria and induced mitochondrial fragmentation (Fig. 3e,f and Supplementary Fig. 5). Treatment with l-aspartate efficiently reversed the reduction in the number of mitochondria and the degree of mitochondrial fragmentation (Fig. 3d–f). Correspondingly, the determination of mitochondrial complex activity and oxidation capacity demonstrated that l-aspartate treatment in mice increased mitochondrial complex I and II activity and increased mitochondrial oxygen consumption, leading to improved ATP production in isolated mitochondria (Fig. 3g–j). Western blot analysis revealed that treatment with l-aspartate increased the expression level of a network of proteins correlated with mitophagy (BNIP3, PINK1 and LC3), mitobiogenesis (TFAM and PGC-1α) and energy expenditure (pLKB1^Ser431^, pAMPK^Thr172^ and CPT-1α) (Fig. 3k). Immunohistochemical analysis confirmed the ability of l-aspartate to induce the expression of the mitobiogenesis regulators, including TFAM and PGC-1α (Fig. 3l).

#### l-aspartate suppresses platelet activation associated with increased cGMP levels

**Fig. 4:**
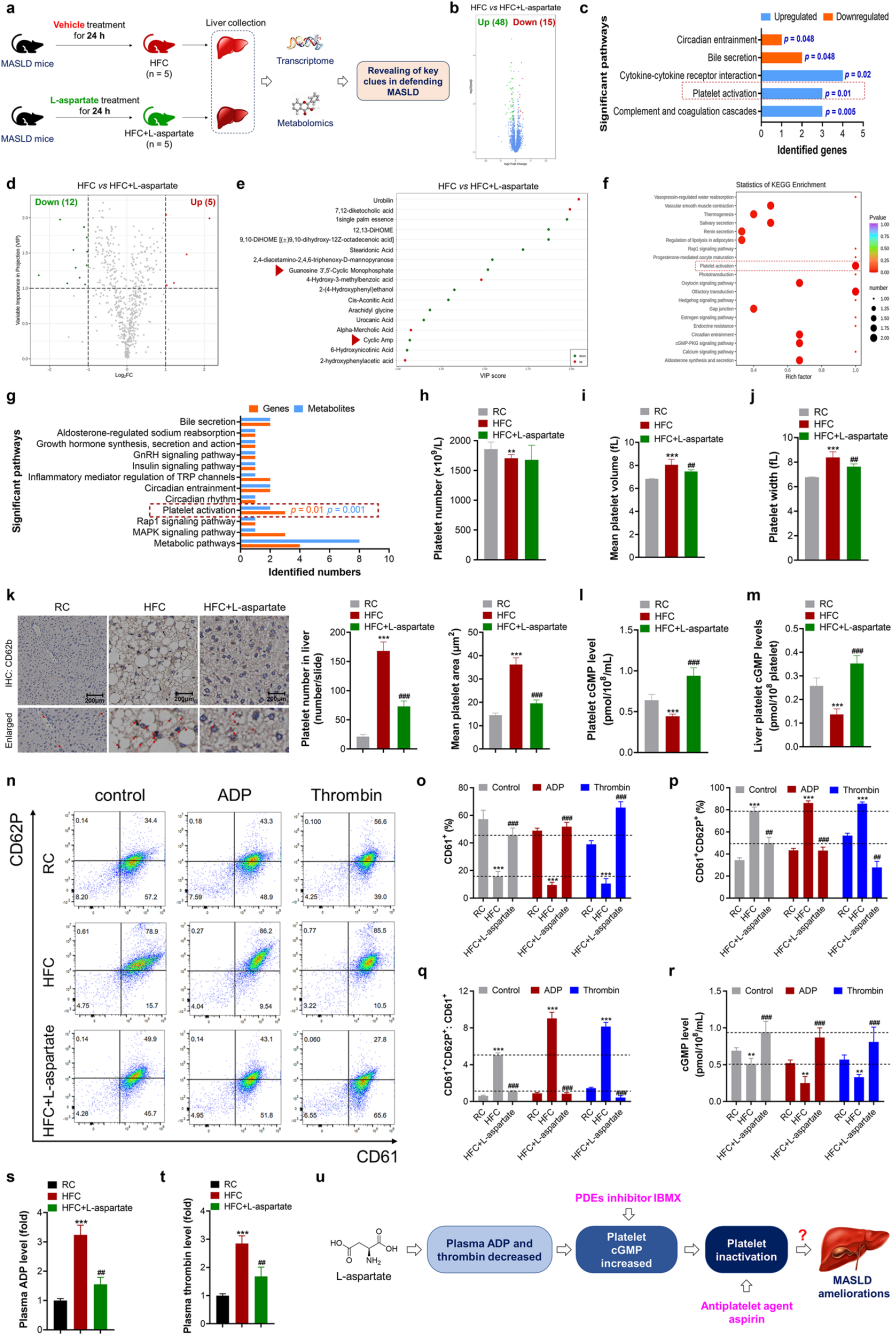
Increased cGMP level in platelets suppresses platelet activation upon l-aspartate treatment. HFC diet feeding-induced MASLD mice (12 weeks HFC diet feeding) were treated with saline or l-aspartate (100 mg/kg) once for 24 h, then livers were isolated for transcriptome and metabolomics analysis. **a** A schematic diagram of a single dose treatment of l-aspartate. **b** A scatter plot of the log_2_ (fold change) generated by the transcriptome analysis. The dots represent differentially expressed genes according to DEseq2 analysis. A total of 63 genes were identified by the following criteria: a fold change (l-aspartate versus vehicle) according to *Q* < 0.05. **c** KEGG annotation of significant genes. **d** Significantly different metabolites were identified based on the criteria of fold ≥2 and a *Q* value equal to or higher than 0.05. The dot represents differentially abundant metabolites. **e** VIP score analysis of significant differentially abundant metabolites. **f** Annotation of the identified significant metabolites. **g** Transcriptome and metabolomics joint analysis. **h**–**j** Determination of plasma platelet number (**h**), volume (**i**) and width (**j**) by flow cytometry assays. **k** Measurement of platelet number in livers by immunohistochemistry analysis of CD42b and quantification. **l**,**m** Determination of cGMP level in platelets isolated from plasma (**l**) or liver (**m**) of RC, HFC or l-aspartate-treated HFC mice after 11 weeks HFC diet feeding with or without L-aspartate treatment. **n**–**q** Determination of the suppression effect of L-aspartate in platelet activation in isolated platelets upon thrombin (0.1 U/ml) or ADP (20 μM) stimulation, the ratio of CD61^+^ (**o**), CD61^+^CD62P^+^ (**p**), and the ratio of CD61^+^CD62P^+^/CD61^+^ (**q**) were calculated. **r** Quantification of cGMP level in platelets. **s**,**t** Plasma ADP (**s**) and thrombin (**t**) measurements. **u** The proposed mechanism controlling cGMP level in platelets. *N* = 10 mice/group. *P < 0.05, **P < 0.01 and ***P < 0.001 versus the RC mice; ^#^*P* < 0.05, ^##^*P* < 0.01 and ^###^*P* < 0.001 versus the HFC control mice.

To reveal the core mechanism by which l-aspartate improved mitochondrial quality in the liver, joint hepatic transcriptome and metabolomics analyses were carried out after 24 h of treatment with a single dose of l-aspartate (Fig. 4a). Principal coordinate analysis (PCA) revealed significant differences in the gene cluster between HFC diet-fed control mice and l-aspartate-treated HFC diet-fed mice, as indicated by the significant differences in the expression of 63 genes, including 15 genes with decreased expression and 48 with increased expression (HFC diet-fed control versus l-aspartate-treated HFC diet-fed mice; Fig. 4b and Supplementary Fig. 6a). These identified genes were annotated according to the Gene Ontology (GO) database. Among metabolism terms, metabolic pathways, biosynthesis of unsaturated fatty acids and fatty acid metabolism were annotated (Supplementary Fig. 6b). According to the KEGG database, cytokine–cytokine receptor interaction, platelet activation, complement and coagulation cascade and bile secretion, were among those terms highly annotated (Fig. 4c). In total, 10 genes were classified into these significant second-level pathways (Supplementary Fig. 6c). With respect to metabolomics, 17 significant metabolites (including 5 upregulated and 12 downregulated) were identified (Fig. 4d,e and Supplementary Table 3). The platelet activation was also annotated (Fig. 4f). Joint omics analysis revealed that the metabolites cAMP and cGMP were correlated with platelet activation (Fig. 4g).

The quantification of plasma platelets revealed that HFC diet feeding markedly induced plasma platelet activation and aggregation, whereas these effects were weakened upon L-aspartate treatment (Fig. 4h–j). Measurement of activated platelets in the liver via quantification of the specific marker CD62b revealed that HFC diet feeding also induced activated platelet aggregation, whereas a reversal of these effects were observed in L-aspartate-treated HFC diet-fed mice (Fig. 4k). Notably, these decreases were correlated with increased cGMP levels in platelets isolated from the plasma or livers of L-aspartate-treated mice compared to those of HFC diet-fed control mice (Fig. 4l,m). Flow cytometry assays further confirmed the suppressive effect of L-aspartate on platelet activation in plasma, as indicated by increases in the number of CD61^+^ platelets and decreases in the number of CD61^+^CD62P^+^ platelets as well as the ratio of CD61^+^CD62P^+^ to CD61^+^ platelets (Fig. 4n–q). ADP and thrombin are well-known platelet activation inducers, and we found that either ADP or thrombin induction further exacerbated HFC diet feeding-induced platelet activation in parallel with reduced cGMP levels in platelets (Fig. 4n). Notably, the platelets isolated from the plasma of L-aspartate-treated HFC diet-fed mice efficiently resisted ADP or thrombin induction, as indicated by decreases in the number of CD61^+^CD62P^+^ platelets and the ratio of CD61^+^CD62P^+^ to CD61^+^ platelets. These effects were correlated with sustained increases in cGMP levels in platelets (Fig. 4r). However, L-aspartate addition had no major effect on suppressing thrombin-induced platelet activation in RC-fed mice and marginally affected the decrease in cGMP levels (Supplementary Fig. 7), suggesting that increasing cGMP levels efficiently resisted platelet activation.

To support the above speculations, we incubated platelets isolated from RC-fed mice with the pan phosphodiesterase (PDE) inhibitor IBMX (which efficiently increases cGMP levels) in the presence of thrombin stimulation and expectedly found that IBMX addition efficiently blocked thrombin-induced platelet activation (Supplementary Fig. 8), implying that the L-aspartate-induced increase in cGMP levels in platelets may not be the direct action of L-aspartate on platelets. Therefore, we determined the effect of L-aspartate on the plasma levels of ADP and thrombin in mice with MASLD and found that L-aspartate treatment efficiently reversed HFC diet-induced increases in the levels of ADP and thrombin in mice with MASLD (Fig. 4s,t). Taken together, these data suggest that L-aspartate suppresses platelet activation via increased cGMP levels through decreasing ADP and thrombin levels in mice with MASLD (Fig. 4u).

#### Inactivation of platelet activation by aspirin ameliorates MASLD in mice

To explore whether the inhibition of platelet activation facilitates MASLD amelioration in vivo, we treated HFC diet-fed mice with MASLD with the approved antiplatelet agent aspirin (1.5 mg/kg bodyweight) once daily for 6 weeks as previously reported^22^ (an animal model is depicted in Supplementary Fig. 9a). Notably, compared to HFC diet feeding, aspirin treatment efficiently decreased HFC diet feeding-induced liver enlargement and heavy hepatic lipid accumulation (Supplementary Fig. 9b,c). Plasma liver enzyme examination revealed significant amelioration of liver injury upon aspirin treatment (Supplementary Fig. 9d). H&E and oil red O staining of the liver further confirmed the ability of aspirin to ameliorate hepatic steatosis and liver injury (Supplementary Fig. 9e).

#### Activated platelets induced mitochondrial fragmentation and lipid accumulation in hepatocytes

**Fig. 5:**
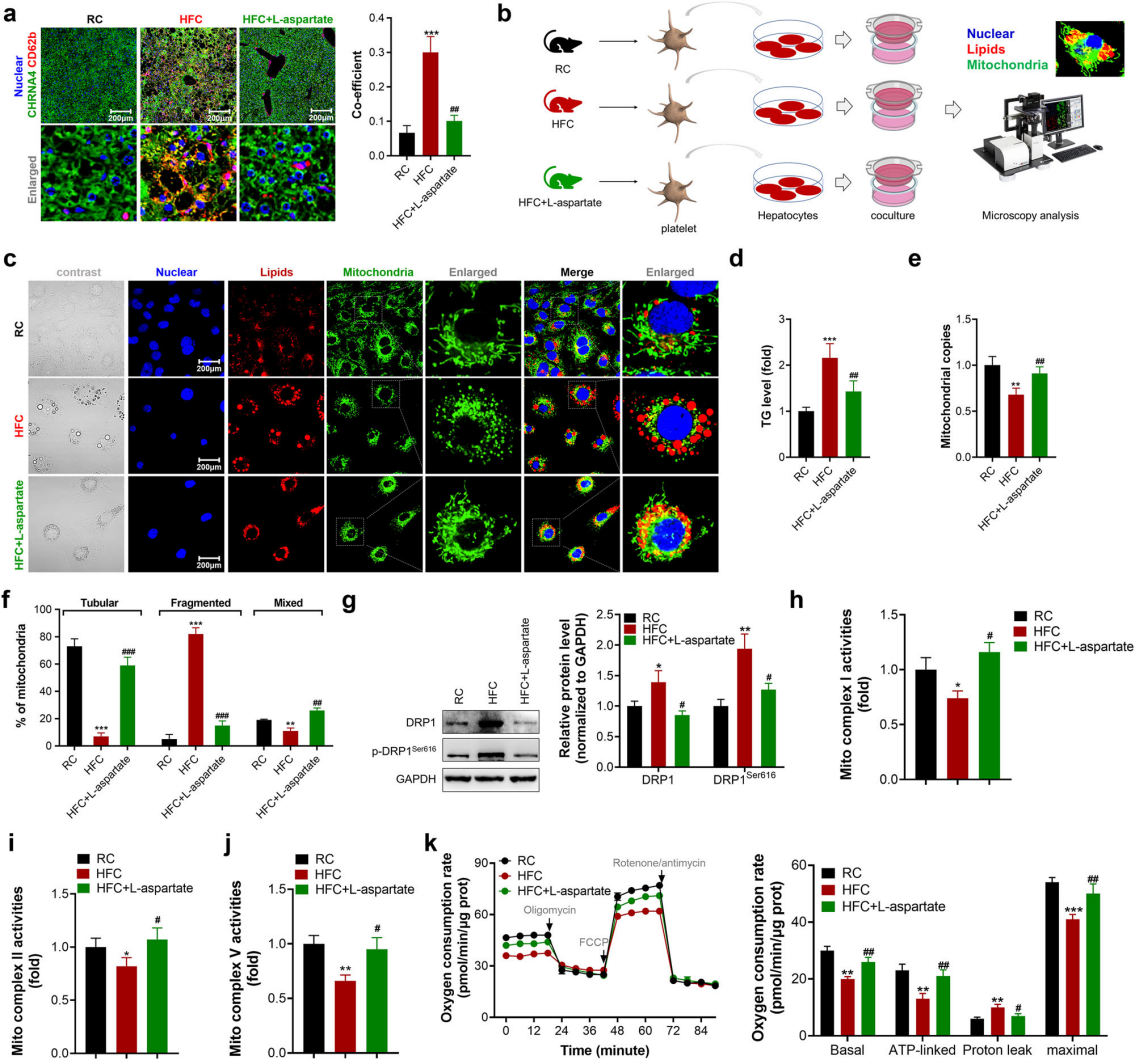
Activated platelets facilitate lipid accumulation and mitochondrial fragmentation in hepatocytes. **a** Imaging of CD62b (activated platelet marker) and CHRNA4 (hepatocyte marker) in the livers of mice (left) and colocalization analysis (right). Scale bar, 100 μm. **b** A schematic diagram of co-incubation of platelets together with hepatocytes. The platelets were isolated from the plasma of RC, HFC or HFC + l-aspartate treated mice. **c** Imaging of lipids and mitochondria in hepatocytes. Scale bar, 50 µm. **d** Quantification of the cellular lipid content. **e** Mitochondrial copies measurement. **f** Mitochondrial morphology analysis and quantification. **g** Determination of DRP1 and its phosphorylation level in hepatocytes (left) and quantification (right). **h**–**j** Measurement of mitochondrial complex I (**h**), II (**i**) and V (**j**) activities. **k** The evaluation of cellular metabolic effect. *N* = 4 independent biological experiments. *P < 0.05, **P < 0.01 and ***P < 0.001 versus the RC mice; ^#^*P* < 0.05, ^##^*P* < 0.01 and ^###^*P* < 0.001 versus the HFC mice.

Although studies have demonstrated that platelet activation is positively correlated with and contributes to the development of MASLD and MASH in both mice and humans^16,23,24^, the role and exact mechanisms underlying platelets in MASLD are less reported. We first analyzed the interactions between activated platelets and hepatocytes by examining the colocalization of their specific markers in the liver and found that HFC diet feeding enhanced the interactions between activated platelets and hepatocytes, as indicated by increased colocalization signals of CD62b and CHRNA4 (hepatocyte marker) (Fig. 5a). Furthermore, we incubated platelets isolated from the plasma of RC-fed, HFC diet-fed and L-aspartate-treated HFC diet-fed mice with hepatocytes and found that incubation of hepatocytes with activated platelets isolated from HFC diet-fed mice resulted in greater lipid accumulation in hepatocytes than did incubation with those from RC-fed mice, as indicated by a TG assay (Fig. 5b–d). Instead, incubation of hepatocytes with platelets isolated from L-aspartate-treated HFC diet-fed mice decreased lipid accumulation. Notably, in addition to severe lipid accumulation, incubation with activated platelets isolated from HFC diet-fed mice decreased mitochondrial dysfunction, as indicated by decreased numbers of mitochondria and an increased degree of mitochondrial fragmentation in hepatocytes (Fig. 5c,e,f). However, these effects were ameliorated when hepatocytes were incubated with platelets isolated from L-aspartate-treated HFC diet-fed mice (Fig. 5c,e,f). Consistent with the observed changes in mitochondrial morphology, incubation of hepatocytes with activated platelets isolated from HFC diet-fed mice increased the expression level of the mitochondrial fission marker DRP1 and its phosphorylation level (Ser616) (Fig. 5g), leading to suppressions of mitochondrial complex I, II and V activity and mitochondrial oxidation capacity (Fig. 5h–k). By contrast, incubation of hepatocytes with platelets isolated from L-aspartate-treated HFC diet-fed mice reversed the increases in the levels of DRP1 and its p-DRP1, thereby reversing the suppression of mitochondrial complex I, II and V activity and the increase in the mitochondrial oxidation capacity. Taken together, these data indicate that activated platelets exacerbate mitochondrial dysfunction and lipid accumulation in hepatocytes involved in the development of MASLD.

#### ATP is a toxic substrate exacerbating OA-induced steatosis and mitochondrial fragmentation in hepatocytes

**Fig. 6:**
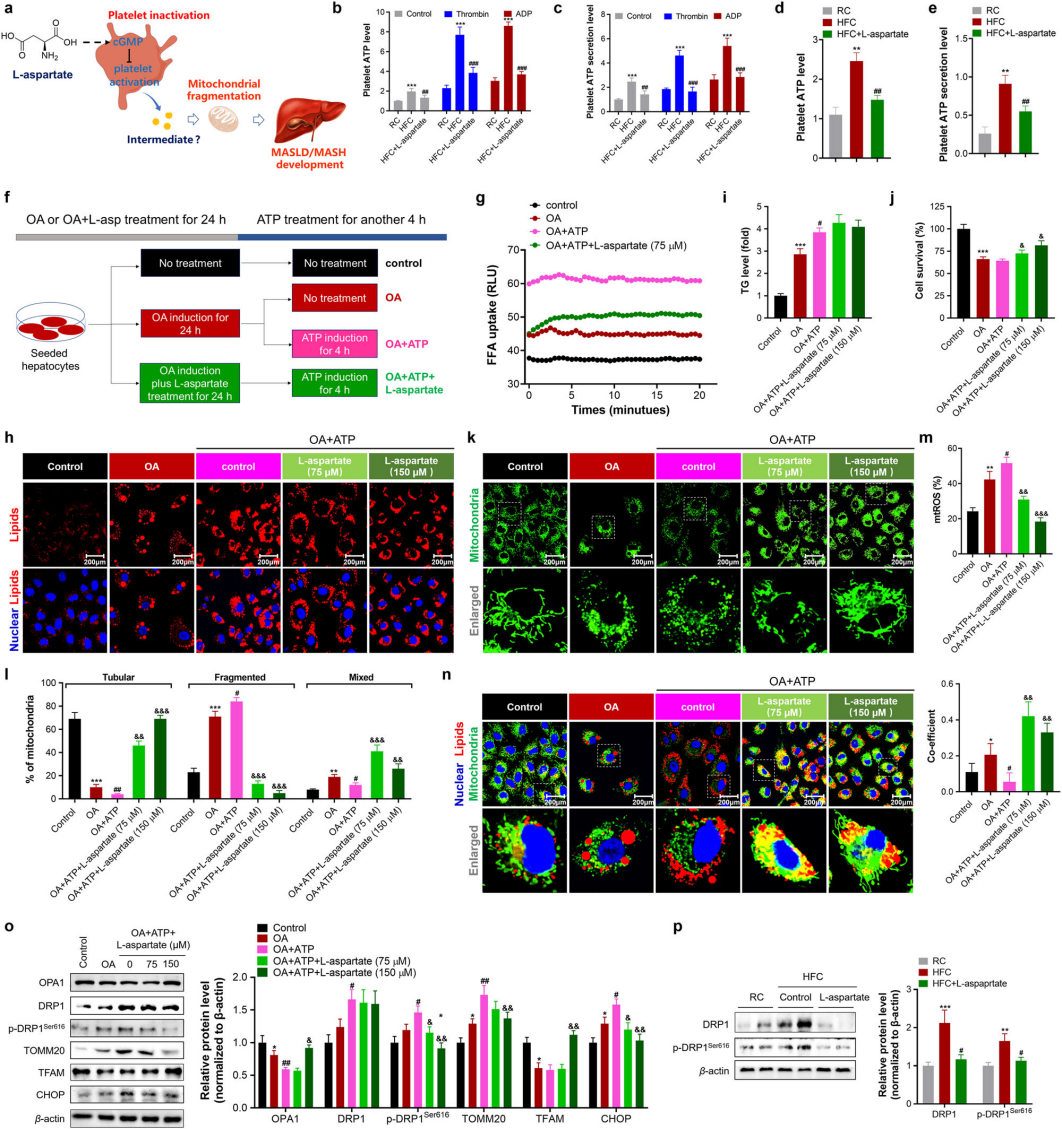
ATP induces lipid accumulation and mitochondrial fragmentation in hepatocytes. **a** A schematic of the hypothesis underlying the mechanism by which L-aspartate counteracts MASLD in mice. **b**,**c** Quantification of ATP production (**b**) and secretion (**c**) levels in platelets isolated from the plasma of mice. **d**,**e** Quantification of ATP production (**d**) and secretion (**e**) levels in platelets isolated from the liver of mice. **f** The experimental diagram of ATP induction in OA-treated hepatocytes. **g** FFA uptake assay. **h** Imaging of lipids in hepatocytes. **i** Cellular TG quantification. **j** Cell survival assay. **k**,**l** Mitochondrial morphology analysis (**k**) and quantification (**l**). **m** mtROS level quantification. **n** Imaging mitochondria and lipids (left) and quantification (right). **o** Examination of proteins related to mitochondrial dynamics, mitobiogenesis and oxidative stress. **p** Expression of DRP1 and p-DRP1 in livers (left) and quantification (right). *N* = 10 mice per group/4 independent biological experiments. *P < 0.05, **P < 0.01 and ***P < 0.001 versus control cells or RC mice; ^#^*P* < 0.05, ^##^*P* < 0.01 and ^###^*P* < 0.001 versus OA-treated hepatocytes or HFC control mice; ^&^*P* < 0.05, ^&&^*P* < 0.01 and ^&&&^*P* < 0.001 versus OA + ATP-treated hepatocytes.

We then explored the mechanism by which activated platelets facilitate lipid accumulation and mitochondrial fragmentation in hepatocytes by determining the secreted metabolites (Fig. 6a). ATP is the main source of energy and a coenzyme that participates in the metabolism of fat, protein, nucleic acid and nucleotides. ATP can be secreted from damaged or necrotic cells and serves as an intermediator that interacts with ATP receptors (for example, P2X2 and P2X4), initiating biological processes such as inflammation and pyroptosis^25,26^. Interestingly, the platelets isolated from the plasma of HFC diet-fed mice presented marked increases in the production and secretion levels of ATP, whereas severe exacerbations were observed with the addition of ADP or thrombin (Fig. 6b,c). The platelets isolated from L-aspartate-treated HFC diet-fed mice presented reduced ATP production and secretion levels and resisted the stimulation of ADP or thrombin (Fig. 6b,c). A similar effect was also observed in platelets isolated from the livers (Fig. 6d,e). Moreover, the suppressive effect was further observed in IBMX- or aspirin-treated platelets isolated from HFC diet-fed mice stimulated with thrombin (Supplementary Fig. 10).

Next, we determined the effects of exogenous ATP on FFA uptake, lipid accumulation and mitochondrial function in hepatocytes with or without OA stimulation; the experimental diagram is depicted in Fig. 6f. Interestingly, ATP addition marginally affected FFA uptake but markedly increased cellular lipid levels in hepatocytes (Supplementary Fig. 11a–c). The addition of ATP to OA-treated cells increased FFA uptake and exacerbated lipid accumulation, leading to greater lipotoxicity, as indicated by fewer surviving cells (Fig. 6g–j). The addition of L-aspartate to hepatocytes treated with ATP alone or OA plus ATP efficiently reversed lipid accumulation and hepatocyte death (Fig. 6h–j and Supplementary Fig. 11b,c). In addition to these changes, ATP addition induced mitochondrial fragmentation in hepatocytes, whereas severe exacerbation occurred with the cotreatment of ATP and OA, as indicated by increases in the ratio of fragmented mitochondria and mitochondrial ROS (mtROS) levels (Fig. 6k–m and Supplementary Fig. 11c,d). L-Aspartate treatment markedly decreased the degree of mitochondrial fragmentation and mtROS levels. In addition, L-aspartate treatment enhanced the colocalization of lipids and mitochondria in cells (Fig. 6n and Supplementary Fig. 11c,d).

Consistent with the observed changes in mitochondrial morphology, western blot analysis revealed that ATP addition exacerbated OA-induced mitochondrial dynamics dysfunction, as indicated by decreases in the level of OPA1 (a mitochondrial dynamin-like GTPase) and increases in DRP1, p-DRP1^Ser616^ and TOMM20 levels (Fig. 6o). Moreover, ATP addition further decreased the levels of TFAM and increased the expression level of CHOP, a representative marker of oxidative stress. L-Aspartate treatment reversed OA + ATP-induced mitochondrial dynamics dysfunction and the suppression of mitogenesis in a dose-dependent manner. These reversals were also observed in the livers of L-aspartate-treated mice (Figs. 3k and 6p), which is consistent with our previous findings^10^.

#### ATP-mediated P2X7–NEK7–DRP1 axis activation contributes to mitochondrial dysfunction, facilitating the progression of MASLD in mice

**Fig. 7:**
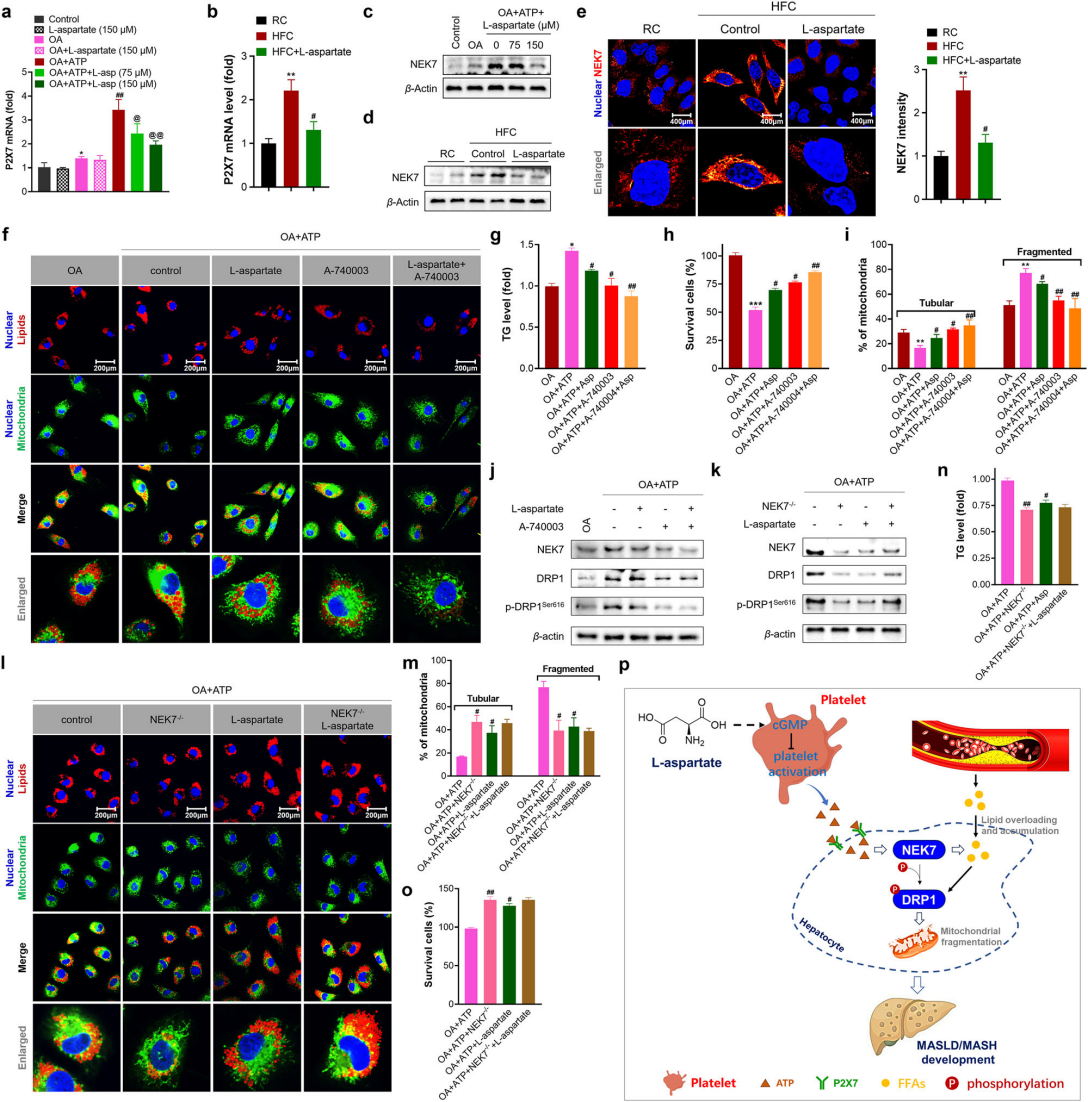
L-aspartate blocks ATP-mediated P2X7–NEK7–DRP1 axis activation. **a** Examination of the P2X7 mRNA level in OA or OA + ATP-treated hepatocytes. *P < 0.05 versus control cells; ^#^*P* < 0.05 and ^##^*P* < 0.01 versus OA-treated hepatocytes; ^@^*P* < 0.05 and ^@@^*P* < 0.01 versus OA + ATP-treated hepatocytes. **b** Examination of the P2X7 mRNA level in the liver. **c**,**d** Examination of the NEK7 protein level in OA or OA + ATP-treated hepatocytes (**c**) or liver (**d**) of mice. **e** Immunofluorescence analysis of NEK7 in the liver (left) and quantification (right). Scale bar, 200 µm. **f**–**j** The effect of the ATP receptor P2X7 inhibitor A-740003 in mitochondrial fragmentation, lipid accumulation and hepatocyte death: imaging of lipids and mitochondria in hepatocytes (**f**) (scale bar, 50 µm), quantification of cellular TG levels (**g**) and cell survival (**h**), mitochondrial morphology measurement (**i**), and the expression of proteins related to the NEK7–DRP1 axis (**j**). **k**–**o** Determination of the role of NEK7 in controlling mitochondrial fragmentation, lipid accumulation and hepatocyte death by knocking down NEK7 in hepatocytes: expression of proteins related to NEK7–DRP1 axis (**k**), imaging lipid and mitochondria in hepatocytes (**l**) (scale bar, 50 µm), mitochondrial morphology measurement (**m**) and the quantification of cellular TG level (**n**) and cell survival (**o**). **p** A schematic of the proposed mechanism by which L-aspartate counteracts MASLD. *N* = 4 independent biological experiments. *P < 0.05, **P < 0.01 and ***P < 0.001 versus the OA-treated hepatocytes; ^#^*P* < 0.05, ^##^*P* < 0.01 and ^###^*P* < 0.001 versus the OA + ATP-treated hepatocytes.

P2X receptors are nonselective cation channels located on the cell membrane that sense extracellular ATP and participate in several biological functions, such as immunity, inflammation and pain^27,28^. We first observed the expression of P2X receptors in OA-treated hepatocytes and found that OA incubation selectively increased the expression level of P2X7, whereas this increase was further enhanced upon ATP addition (Fig. 7a). Notably, other P2X receptors, including P2X2 and P2X4, showed no major increases upon treatment with either OA or OA plus ATP (Supplementary Fig. 12). Notably, among these receptors, L-aspartate treatment decreased only the transcription level of P2X7. In addition, L-aspartate treatment reversed HFC diet-induced increases in the expression of P2X7 in the liver (Fig. 7b). Intriguingly, L-aspartate treatment marginally affected the transcription level of P2X7 in control or OA-treated hepatocytes.

NIMA-related kinase (NEK) serves as a downstream effector of P2X7^29^, and activation of the P2X7–NEK axis involves not only inflammasome activation through interactions with NLRP3 but also mitochondrial dynamics through interactions with OPA1 and DRP1^30,31^. We found that both OA treatment and HFC diet feeding activated NEK7, as indicated by increases in the expression levels of NEK7, while severe exacerbation was observed upon ATP addition compared to that in OA-treated hepatocytes (Fig. 7c,d). Instead, L-aspartate treatment of either OA + ATP-treated hepatocytes or HFC diet-fed mice efficiently reversed the activation of NEK7. Immunofluorescence analysis also confirmed the suppressive effect of L-aspartate on the hepatic levels of NEK7 (Fig. 7e).

To further confirm that the suppression of P2X7–NEK7 activation mediated by L-aspartate treatment was correlated with the anti-MASLD effect of L-aspartate in mice, we treated OA + ATP-induced hepatocytes with the P2X7 inhibitor A-740003 or L-aspartate alone and together and found that, similar to L-aspartate, A-740003 decreased the cellular TG level and hepatocyte death (Fig. 7f–h). These effects were correlated with attenuation of mitochondrial fragmentation and suppression of the NEK7–DRP1 axis (Fig. 7i, j). Additional effects were observed when L-aspartate was combined with A-740003, suggesting that P2X7 is an upstream regulator of the NEK7–DRP1 axis. Furthermore, we deleted NEK7 in hepatocytes (Supplementary Fig. 13) and found that deletion of NEK7 decreased the total and phosphorylated levels of DRP1, leading to attenuations of mitochondrial fragmentation and lipid accumulation, thereby reducing hepatocyte death (Fig. 7k–o). Adding L-aspartate in OA + ATP-treated NEK7^−/−^ hepatocytes showed no additive effects on the suppression of mitochondrial fragmentation, lipid accumulation or hepatocyte death compared to treatment alone. On the contrary, overexpressed NEK7 (NEK7^+/+^) in hepatocytes greatly exacerbated OA + ATP-induced DRP1 activation and mitochondrial fragmentation (Supplementary Fig. 14a,b). These effects were noted alongside more severe lipid accumulation and hepatocyte damage (Supplementary Fig. 14c–e). Adding L-aspartate to the OA + ATP-treated NEK7^+/+^ hepatocytes significantly reversed NEK7–DRP1 axis activation and followed mitochondrial fragmentation in parallel with lipid accumulation and hepatocyte damage ameliorations. Moreover, treatment with the antiplatelet agent aspirin blocked ATP + OA-induced mitochondrial fragmentation, lipid accumulation and hepatocyte death (Supplementary Fig. 15). Taken together, these data indicate that the P2X7–NEK7–DRP1 axis is closely related to ATP-mediated mitochondrial fragmentation, lipid accumulation and cell death in hepatocytes and that L-aspartate counteracts MASLD/MASH in mice by rejuvenating mitochondria through blocking platelet activation-mediated ATP release followed by P2X7–NEK7–DRP1 axis activation (Fig. 7p).

## Discussion

As exemplified by the approved anti-MASH agent, enhancing mitochondrial oxidation is widely recognized as a practical strategy for treating MASLD and its progressive diseases, including MASH and hepatic fibrosis^13,32^. The increase in the level of L-aspartate in the liver improved mitochondrial oxidation capacity, thereby counteracting liver fibrosis^12,33^; these findings suggest that L-aspartate is a novel metabolic regulator and potentially useful for treating MASLD and MASH. In the present study, we revealed that plasma and hepatic L-aspartate levels were decreased in mice and humans with MASLD. Using dietary approaches to determine L-aspartate availability, we provide evidence for the causative and therapeutic roles of L-aspartate against MASLD and MASH in mice. These effects are correlated with enhanced whole-body metabolic effects and improved mitochondrial quality in the liver. Mechanistically, cGMP-correlated platelet activation was correlated with the beneficial metabolic effects of L-aspartate. Notably, platelet activation-mediated ATP release followed by P2X7–NEK7–DRP1 axis activation in hepatocytes has been shown to play crucial roles in inducing mitochondrial fragmentation, lipid accumulation and hepatocyte death. Our study revealed the interactions between activated platelets and hepatocytes in driving hepatic mitochondrial dysfunction during MASLD. Moreover, our study systematically explored the therapeutic efficacy of L-aspartate against MASLD and MASH in mice, providing detailed clues for broadening its potential applications in the clinic, especially as ideal anti-MASLD agents.

The accumulation of fatty acids such as TG in the liver is the main manifestation of MASLD. Mitochondria are essential organelles that oxidize lipids, maintaining energy homeostasis and the oxidative/redox balance. Upon lipid flux into hepatocytes, fatty acid β-oxidation is activated but is not enough to compensate for the increase in lipids; instead, lipid peroxidation is induced, resulting in the production of ROS and the activation of oxidative stress and mitochondrial dysfunction^34^. This process leads to a range of physiological abnormalities and the generation of toxic substances such as ATP, mtROS and mtDNA, resulting in cell death and an inflammatory response. Recently, it has been widely acknowledged that mitochondrial dysfunction is closely associated with MASLD^35^. Targeting mitochondrial dysfunction has great potential for reversing liver disease progression^36^. Many approaches have been reported to combat MASLD, including limiting caloric intake and/or increasing the consumption of calories, antidiabetic drugs (that is, metformin), bile acid regulators (that is, obeticholic acid) and antioxidants (that is, vitamin E)^37–40^. Furthermore, some naturally occurring compounds, such as capsaicin, HN-002, resveratrol, berberine and quercetin, reportedly enhance mitochondrial function to ameliorate MASLD^15,41–43^. In the present study, L-aspartate treatment improved whole-body metabolism and increased energy expenditure in mice. In the liver, L-aspartate treatment reversed HFC diet-induced decreases in mitochondrial quality and suppressed the expression of metabolism-related markers, which contributed to the anti-MASLD effects of L-aspartate in mice.

L-aspartate serves as a shuttle carrier of the TCA cycle and is widely used as a liver-protecting agent owing to its ability to regulate nitrogen and urea metabolism; however, whether other mechanisms are involved cannot be excluded, and this possibility has previously not been studied. Recently, L-aspartate has been proposed to be a promising therapeutic candidate for MASLD and MASH^44,45^. Our previous and latest studies explored the metabolic regulatory effects of L-aspartate on mitochondrial oxidation in different liver disease models and demonstrated the feasibility of L-aspartate in curbing MASLD/MASH. In the present study, we explored the anti-MASLD and anti-MASH effects of L-aspartate in relevant mouse models and found that L-aspartate treatment efficiently corrected all manifestations of MASLD and MASH, including steatosis, inflammation, liver injury and fibrosis. These therapeutic actions appear to largely fulfill the criteria essential for the treatment of MASLD and MASH^46^. Considering the higher dosage of L-aspartate used and the long duration of drug intervention, we believe that the key mechanism underlying L-aspartate-regulated mitochondrial function still needs to be explored.

By performing hepatic transcriptome and metabolomics joint analysis, the metabolite cGMP and platelet activation were highly annotated upon treatment with a single dose of L-aspartate. Platelets are the smallest blood cells; upon activation, they are involved not only in arterial thrombosis but also in other physiological and pathophysiological processes. Many studies have shown that platelets not only regulate liver homeostasis and function but also participate in liver pathobiology^47^. Platelets interact with sinusoidal endothelial cells and facilitate the infiltration and recruitment of lymphocytes^48^; they can also interact with hepatic macrophages (Kupffer cells) to regulate liver injury, MASH and fibrogenesis^49^. Recently, the role of platelets in MASLD/MASH development has gained recognition. Accumulating evidence suggests that activated platelets are significant contributors to MASLD/MASH. Patients with MASLD have increased numbers of platelets and larger sizes of platelets, which reflect the severity of inflammation and fibrosis in the liver^16,50–52^. Furthermore, the use of antiplatelet therapy alleviated MASH development and reduced liver damage in patients with MASLD^51,53^. In the present study, L-aspartate treatment reduced the number of platelets and their aggregation in the liver. This blocking effect was further verified in isolated platelets even under stimulation with ADP or thrombin. This suppression was associated with increases in the level of cGMP, an identified negative regulator of platelet activation^54,55^. This suppression was also reconfirmed by the PDE inhibitor IBMX. An unexpected finding of our current study was that incubating platelets isolated from HFC diet-fed mice with hepatocytes facilitated lipid accumulation alongside mitochondrial fragmentation in hepatocytes, which are key manifestations and initial factors of MASLD. However, reversal effects were observed when platelets isolated from L-aspartate-treated HFC diet-fed mice were incubated, which was in accordance with the in vivo findings. Moreover, treatment with the antiplatelet agent aspirin in mice with HFC diet-induced MASLD efficiently reversed MASLD, indicating that platelet activation plays an essential role in facilitating MASLD, that increased cGMP levels suppress platelet activation and blocked the interaction between activated platelets and hepatocytes contribute to the anti-MASLD effect of L-aspartate. This new finding reveals a direct role of platelets in driving MASLD and metabolic regulation in the liver through their interaction with hepatocytes, which is distinct from previous findings^16^.

Upon activation, platelets undergo a metabolic phenotype switch from mitochondrial respiration to glycolysis as the mitochondria undergo fission and are smaller, causing an imbalance of the reduction/oxidation state and the production and release of toxic substrates^55,56^, including serotonin (5-HT)^57^ and thromboxane A2 (T_X_A2)^58^ and so on; however, the effects of these metabolites on lipid metabolism and mitochondrial function regulation are unreported. ATP, an energy substrate produced mainly through mitochondrial oxidation, is released by activated platelets during inflammation^59^. Studies have demonstrated that increased ATP release from the platelets of patients with diabetes contributes to an increased risk of vascular complications^60^. In the present study, both HFC diet feeding and ADP or thrombin stimulation significantly increased ATP release in platelets, which binds the P2X7 receptor, leading to downstream NEK7–DRP1 axis hyperactivation and contributing to mitochondrial fragmentation and lipid storage in hepatocytes. ATP addition further exacerbated OA-induced P2X7–NEK7–DRP1 axis activation. Similarly, activation of the P2X7–NEK7–DRP1 axis occurred in the liver upon HFC diet feeding. Instead, blockade of P2X7, knockdown of NEK7 or treatment with the antiplatelet agent aspirin efficiently suppressed ATP-induced metabolic toxic effects in hepatocytes. In addition, treatment with L-aspartate in OA + ATP-treated hepatocytes or HFC diet-induced mice inhibited the P2X7–NEK7–DRP1 axis, highlighting the crucial role of the ATP–P2X7–NEK7–DRP1 axis in regulating mitochondrial homeostasis and hepatocyte survival. Intriguingly, L-aspartate treatment marginally affected P2X7 level in hepatocytes or OA-treated hepatocytes, further pinpointing the specific role of L-aspartate in blocking ATP signaling driven by activated platelets.

MASLD has become the world’s leading cause of liver disease and there is an urgent need to develop safe and effective therapeutic agents. Our previous studies confirmed the therapeutic effects of L-aspartate against MASLD and liver fibrosis^10,12^. Considering that its clinical application in treating liver diseases is highly safe, exploring the potential application of L-aspartate for the treatment of MASLD and MASH (and even liver fibrosis) in humans is important. In addition, L-aspartate is inexpensive, making it a suitable and promising candidate for treating MASLD and MASH. However, the physiological effects of L-aspartate, such as neuronal excitatory and toxic effects, vary, so the effective dose range of L-aspartate for treating MASLD and MASH in humans needs further exploration to avoid side effects. Like THR-β agonists, L-aspartate shares similar metabolic regulation, but its anti-MASLD/MASH mechanism needs further exploration.

In summary, our studies revealed negative correlations between hepatic L-aspartate levels and MASLD in both mice and humans. L-aspartate supplementation efficiently reversed MASLD and MASH in mice and improved whole-body metabolic and hepatic mitochondrial quality. These beneficial effects are driven by increased cGMP levels in platelets, leading to the inhibition of platelet activation and its derived ATP-mediated P2X7–NEK7–DRP1 axis in hepatocytes.

## Supplementary information

Supplementary Information

## Data Availability

All data needed to evaluate the conclusions in the paper are present in the paper and/or the Supplementary Materials. Additional data related to this paper may be requested from the authors.
