# Supplementary material for: Supplementation of L-aspartate corrects MASLD and MASH in mice by inhibiting platelet–hepatocyte interaction-mediated mitochondrial fragmentation via the ATP–P2X7–NEK7–DRP1 axis
Source: Exp Mol Med. 2026 Feb 13;58(2):533–47. doi: 10.1038/s12276-026-01648-9 (PMC12992609; doi:10.1038/s12276-026-01648-9)

Supplementary Information

for

Supplementation of L-aspartate corrects MASLD and MASH in mice by inhibiting platelet-hepatocyte interaction-mediated mitochondrial fragmentation *via* the ATP-P2X7-NEK7-DRP1 axis

Wen-Jie Cao[a], Rui Su[a], Hui-Ling Fu[a], Jun-Jie Wu[a], Lin-sheng Huang[b], Fei-fei Liu[a], Jin Liu[a], Zhong-Ping Jiang[a], Cong-Jun Xu[a], Yong Rao[a, *], Ling Huang[a, *]

[a] *Key Laboratory of Tropical Biological Resources of Ministry of Education, School of Pharmaceutical Sciences, Hainan University, Haikou 570100, China*

[b] *Department of Hepatopancreatobiliary Surgery, Taihe Hospital, Shiyan, 442000, China*

*** Correspondence**

Professor Yong Rao, School of Pharmaceutical Sciences, Hainan University, Haikou 570100, China. E-mail: raoyong@hainanu.edu.cn (Y. Rao).

Professor Ling Huang, School of Pharmaceutical Sciences, Hainan University, Haikou 570100, China. E-mail: Linghuang@hainanu.edu.cn (L. Huang).

Methods

Serum chemical levels detection and liver pathological examinations

The serum and livers were collected, and serum levels of alkaline phosphatase (ALP), glutamic-pyruvic transaminase (ALT) and aspartate aminotransferase (AST) were determined using plasma chemical analyzer.

Hepatic TG levels quantification

Liver tissue or hepatocytes were homogenized and extracted with equal volumes of chloroform/methanol. The chloroform phase was removed to a new tube and dried and was then resuspended in isopropyl alcohol as a total lipid extract sample. The quantities of TG levels were determined according to the manufacturer's protocol (Jiancheng Bio Cat# A110-2, Nanjing, China).

TEM analysis and quantification

Liver sections were fixed in 2% glutaraldehyde in 0.1 M sodium cacodylate buffer (pH 7.4) at 4 °C overnight and post-fixed with 1% osmium tetroxide/1% potassium ferrocyanide for 1 h at room temperature. After fixation, livers were stained then block with 5% aqueous uranyl acetate overnight at room temperature, dehydrated and embedded in Taab epoxy resin. Ultrathin sections were stained with lead citrate and recorded using a Mega view 3 digital camera and iTEM software in a Jeol 100-CXII electron microscope. The number and size of mitochondria were quantified using Image J software.

Hepatic transcriptome analysis

RNA-seq analysis was performed by LC-Biotechnology (Hangzhou, China). RNeasy mini kit (Qiagen, Germany) was used to extract total RNA from livers of mice. Gene abundance was expressed as fragments per kilobase of exon model per million mapped fragments (FPKM). R package edge R was used to analyze the different expressions of mRNA, and the differentially expressed RNAs with |log2(FC)| value >1 and Q value

<0.05 were retained for further analysis. Pathway and process enrichment analysis in Metascape was used to gain further insight into biology behind the differentially expressed RNAs by computing overlaps with WikiPathways gene sets (https://metascape.org/gp/index.htm l#/main/step1).

Hepatic metabolomics analysis

The metabolites in the livers were extracted with 50% methanol Buffer. 20 μL of sample was extracted with 120 μL of precooled 50% methanol, vortexed for 1 min, and incubated at room temperature for 10 min; the extraction mixture was then stored overnight at −20 °C. After centrifugation at 4,000 *g* for 20 min, the supernatants were transferred into new 96-well plates for LC-MS analysis. In addition, pooled QC samples were also prepared by combining 10 μL of each extraction mixture. These samples were then subjected to LC-MS system followed machine orders. A high-resolution tandem mass spectrometer TripleTOF5600 plus (SCIEX, UK) was used to detect metabolites eluted from the ACQUITY UPLC BEH Amide column (100 mm × 2.1 mm, 1.7 μm, Waters, UK). The Q-TOF was operated in both positive and negative-ion modes. The curtain gas was set 30 PSI, Ion source gas1 was set 60 PSI, Ion source gas2 was set 60 PSI, and an interface heater temperature was 650 °C. For positive ion mode, the ionspray voltage floating were set at −4500 V, respectively. The mass spectrometry data were acquired in IDA mode. The TOF mass range was from 60 to 1200 Da. The survey scans were acquired in 150 millisecond and as many as 12 product ion scans were collected if exceeding a threshold of 100 counts per second (counts/s) and with a 1+ charge-state. Total cycle time was fixed to 0.56 s. Four-time bins were summed for each scan at a pulser frequency value of 11 kHz through monitoring of the 40 GHz multichannel TDC detector with four-anode/channel detection. Dynamic exclusion was set for 4 s. During the acquisition, the accuracy was calibrated every 20 samples. Furthermore, to evaluate the stability of the LC-MS during the whole acquisition, a quality control sample (Pool of all samples) was acquired after every 10 samples.

cGMP level quantification

The liver homogenate or platelet lysate was centrifuged at 12 000 *g* at 4 °C for 15 min, the supernatant was isolated for cGMP level quantification according to the instructions of the cGMP ELISA kits (Elabscience, E-EL-0083). The contents of cGMP were calculated by normalizing protein level or platelet numbers.

ATP production measurement

ATP production in isolated mitochondria was measured using the BioVision ATP determination Kit according to manufacturer's instruction.

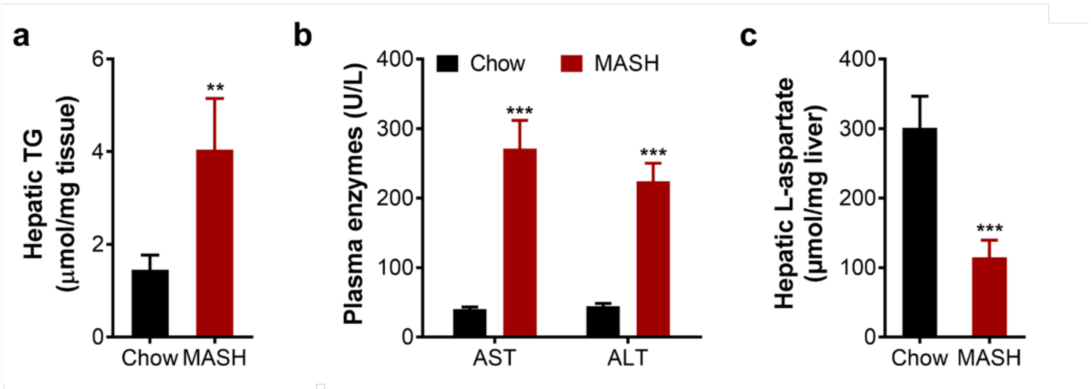

Supplementary Fig. 1. Determination of hepatic L-aspartate level in HFC/MCD diet-induced MASH mice. Male C57BL/6J mice (8- week-old) were fed an HFC diet for 6 weeks and then switched to an MCD diet for another 8 weeks, livers and plasma were collected for indicated analysis. (a) Quantification of hepatic TG content. (b) Measurement of plasma AST and ALT levels. (c) Quantification of hepatic L-aspartate contents. n = 5 mice per group. *** $p < 0.001$, compared with chow-fed mice.

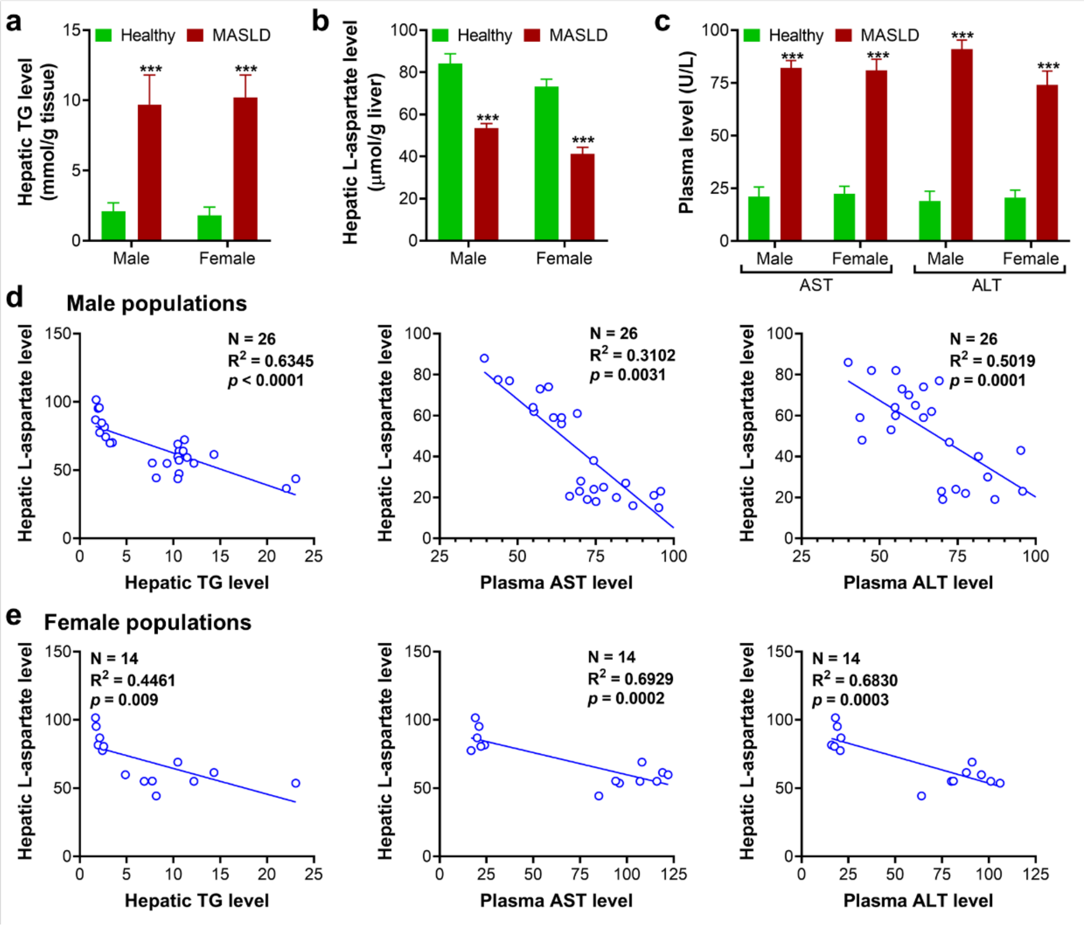

Supplementary Fig. 2. Determination of hepatic L-aspartate level in male and female MASLD populations. 40 human plasma and liver samples (26 male and 14 female) were collected for indicated analysis. (a) Quantification of hepatic TG contents. (b) Quantification of hepatic L-aspartate contents. (c) Measurement of plasma AST and ALT levels. (d) Correlation analysis between hepatic L-aspartate and hepatic TG, plasma AST and ALT in male populations. (e) Correlation analysis between hepatic L-aspartate and hepatic TG, plasma AST and ALT in female populations. n = 40 human populations. *** $p < 0.001$, compared with healthy populations.

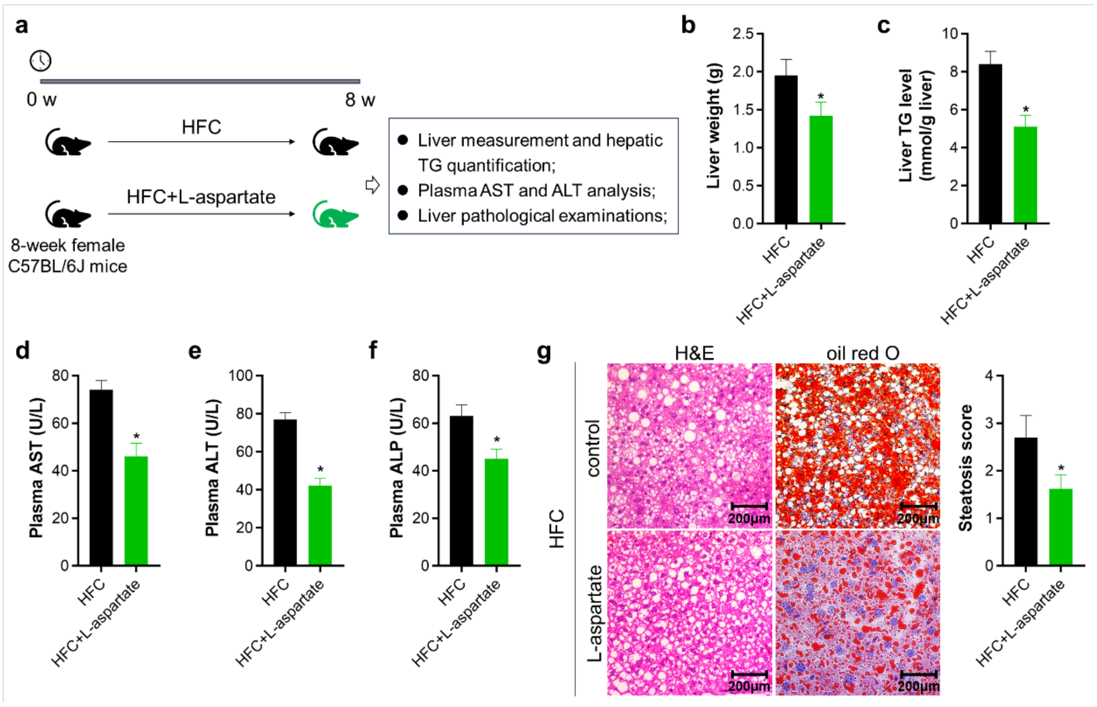

Supplementary Fig. 3. Evaluation of the anti-MASLD effects of L-aspartate in HFC diet-induced female mice. Female C57BL/6J mice (8- week-old) were fed an HFC diet with or without L-aspartate treatment for 8 weeks, livers and plasma were collected for indicated analysis. (a) Schematic diagram of L-aspartate treatment in mice. (b) Liver weight measurement. (c) Quantification of hepatic TG content. (d-f) Measurement of plasma AST, ALT, and ALT levels. (g) H&E and oil-red O staining in liver. Scale bar, 200 μm. n = 5 mice per group. * $p < 0.05$, ** $p < 0.01$, compared with HFC diet-fed mice.

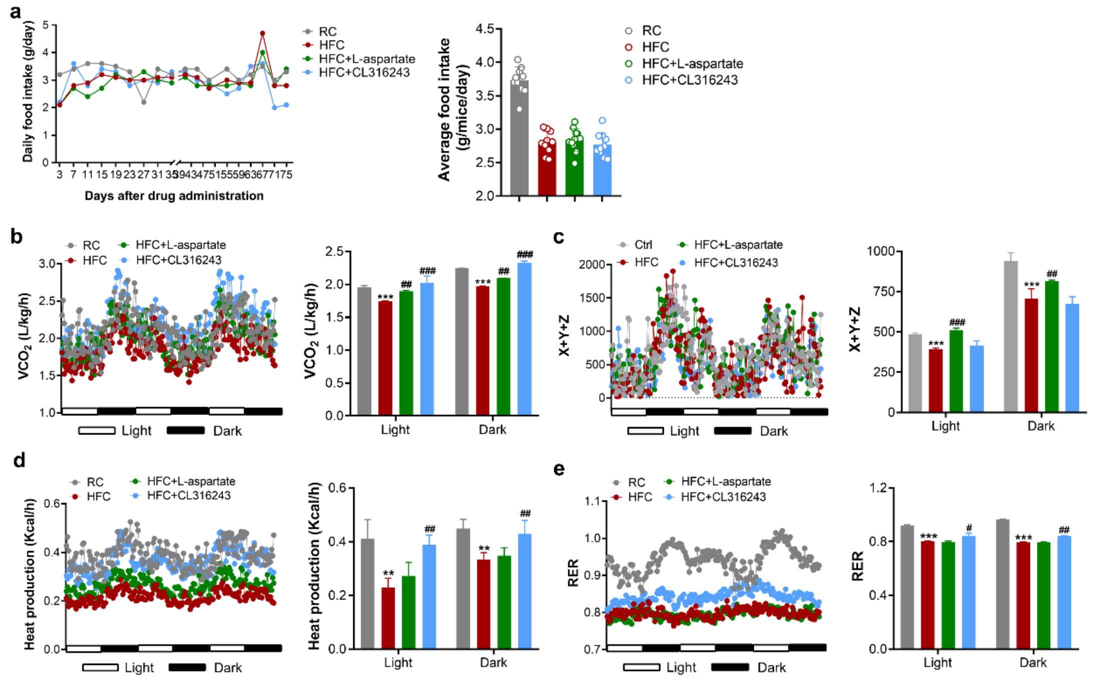

Supplementary Fig. 4. L-aspartate improved whole-body metabolic in HFC diet feeding induced MASLD mice. The HFC diet feeding induced MASLD mice (after 12 weeks HFC diet feeding) were treated with saline, L-aspartate (100 mg/kg), and CL316243 (0.5 mg/kg) for 76 days, the whole-body metabolic efficacy was measured using the CLAMS system. (a) Mouse daily food intake measurement. (b) Determination of carbon dioxide (VCO_2). (c) Physical activity measurement. (d) Heat production measurement. (e) Determination of RER. N = 10 mice/group. * $p < 0.05$, ** $p < 0.01$, *** $p < 0.001$, *vs* the regular chow (RC) mice; $^{\#}$ $p < 0.05$, $^{\#\#}$ $p < 0.01$, $^{\#\#\#}$ $p < 0.001$, *vs* the HFC control mice.

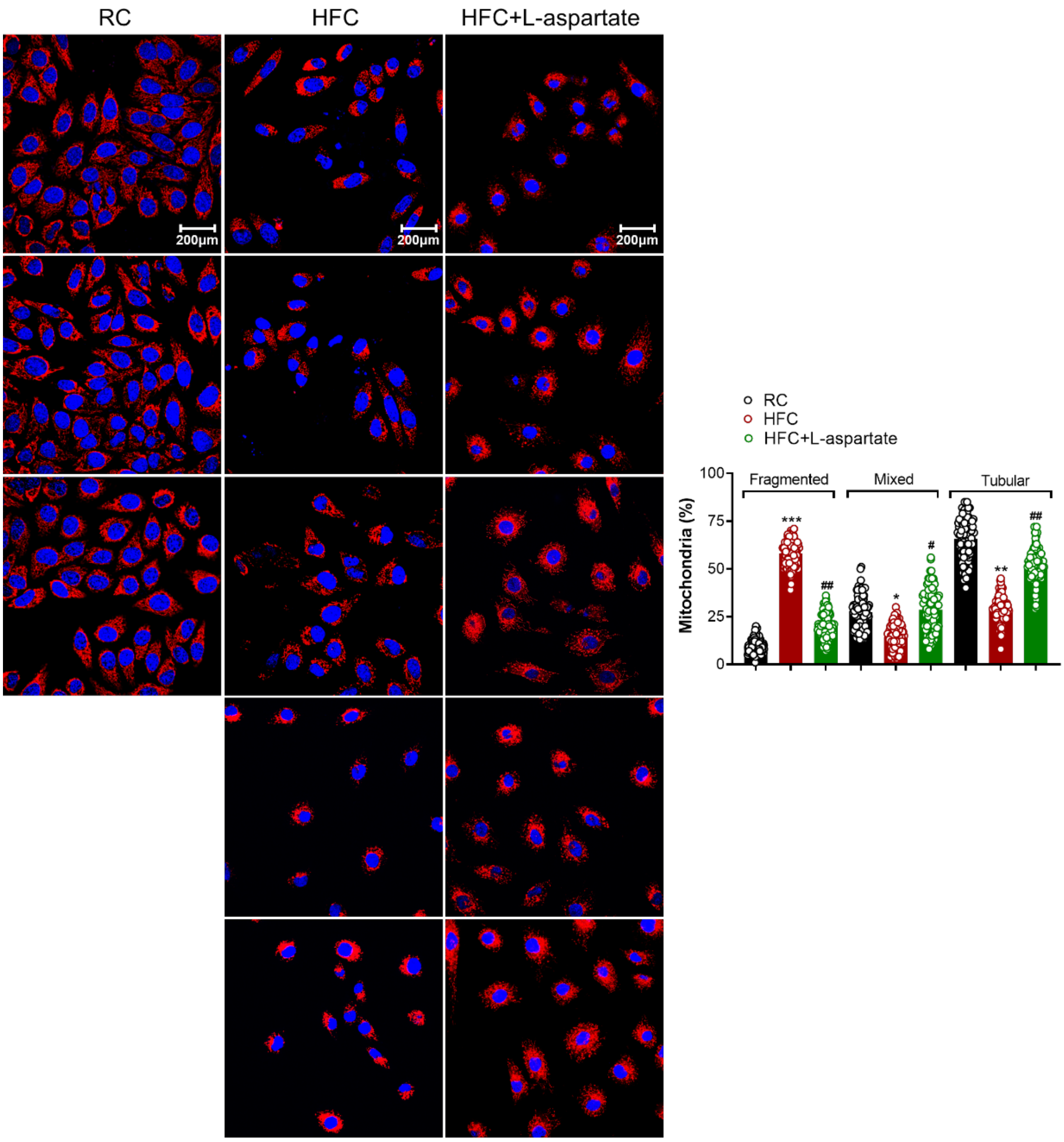

Supplementary Fig. 5. Mitochondrial morphology analysis and quantification in hepatocytes isolated from livers of RC, HFC, and L-aspartate treated HFC mice. 3 mice in each group were randomly selected for hepatocytes isolation and mitochondrial morphology analysis. Data are mean values from 3~5 images and 100 cells. The same cells were used to measure mitochondrial morphology. * $p < 0.05$, ** $p < 0.01$, *** $p < 0.001$, *vs* the regular chow (RC) mice; $^{\#}$ $p < 0.05$, $^{\#\#}$ $p < 0.01$, $^{\#\#\#}$ $p < 0.001$, *vs* the HFC control mice.

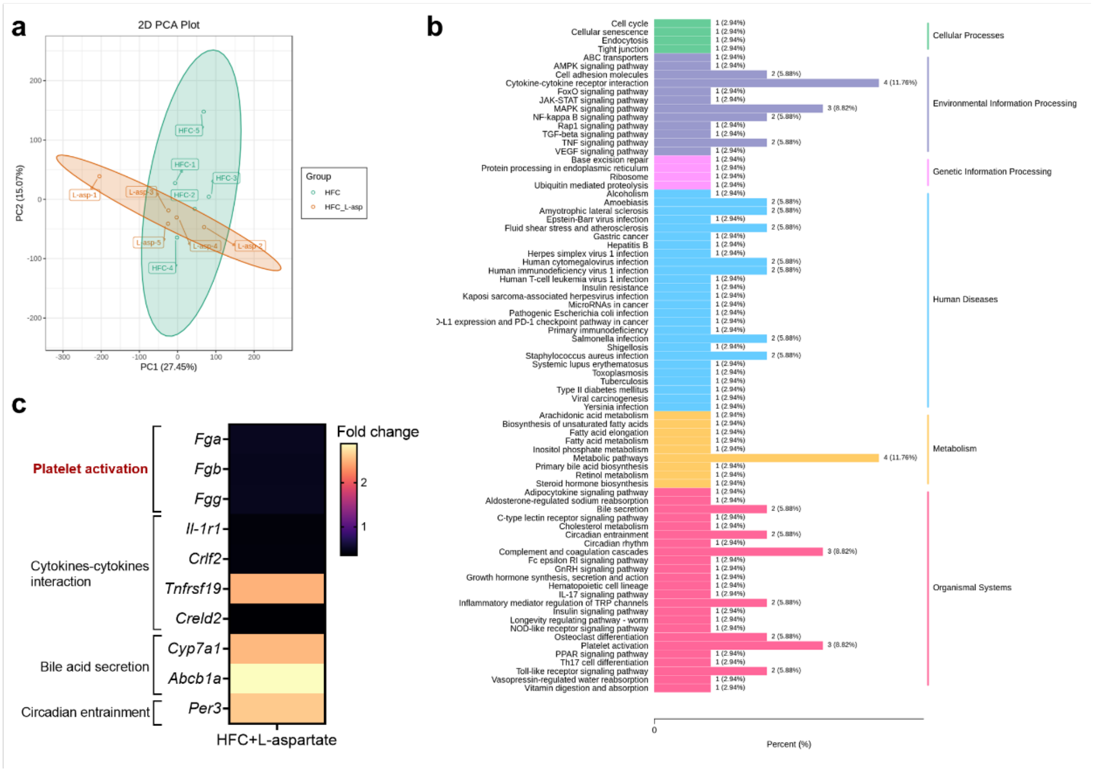

Supplementary Fig. 6. Identification of significant genes in liver upon L-aspartate treatment by transcriptome analysis. N = 5mice/group. (a) PCA analysis of liver samples. (b) Annotation of significant genes by GO analysis. (c) Significant genes correlated with platelet activation, cytokines-cytokines interaction, bile acid metabolism, and circadian entrainment. N = 5 samples/group.

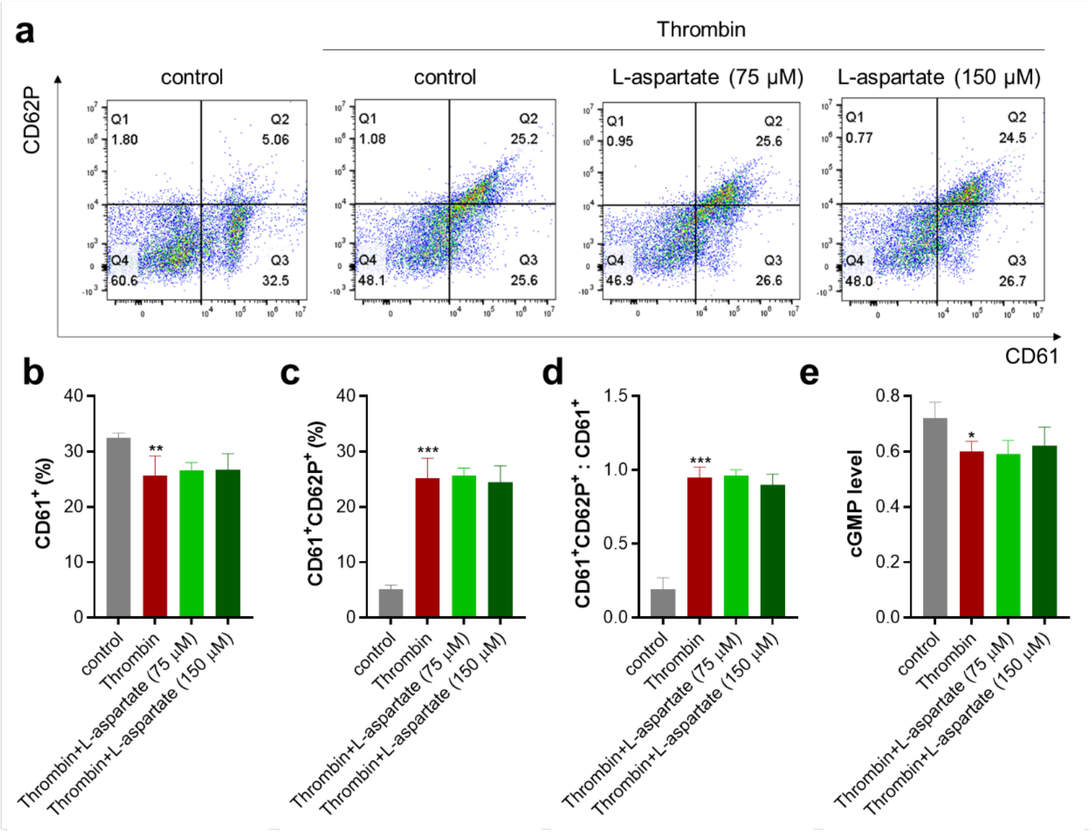

Supplementary Fig. 7. L-aspartate inhibits thrombin induced platelet activation *in vitro*. (a-d) Determination of effect of L-aspartate in suppressing platelet activation induced by thrombin and Quantification. (e) cGMP level quantification. N = 4 independent biological experiments. * $p < 0.05$, ** $p < 0.01$, *** $p < 0.001$, *vs* the control group.

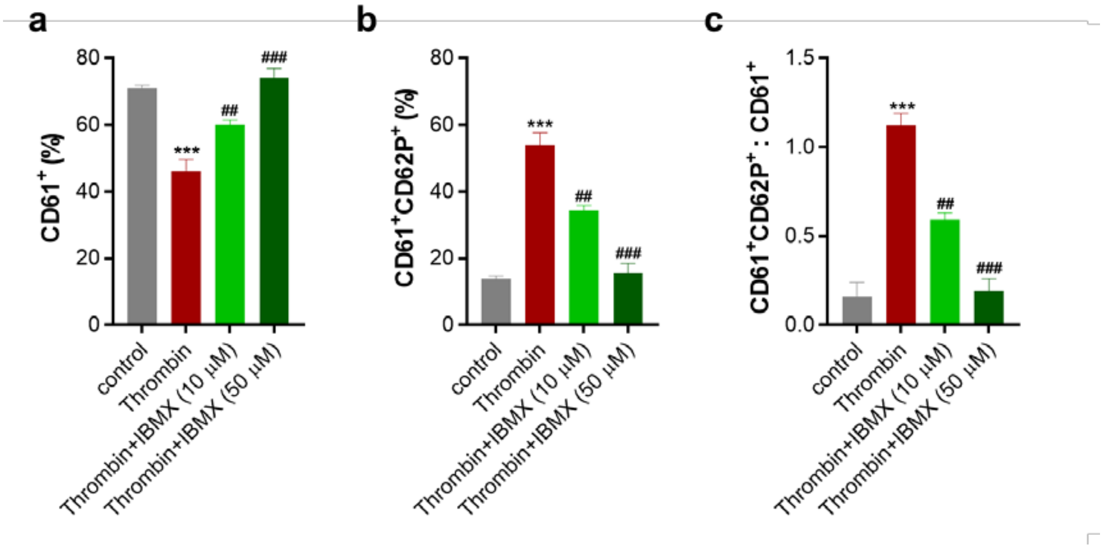

Supplementary Fig. 8. Pan PDE inhibitor IBMX inhibits platelet activation induced by thrombin. (a) Ratio of platelet labelled by CD61 ($CD61^+$). (b) Ratio of activated platelet labelled by CD61 and CD62P ($CD61^+$CD62P+). (c) Ratio of $CD61^+CD62P^+/CD61^+$. N = 4 independent biological experiments. * $p < 0.05$, ** $p < 0.01$, *** $p < 0.001$, *vs* the control group; $^{\#\#}$ $p < 0.01$, $^{\#\#\#}$ $p < 0.001$, *vs* the thrombin treated group.

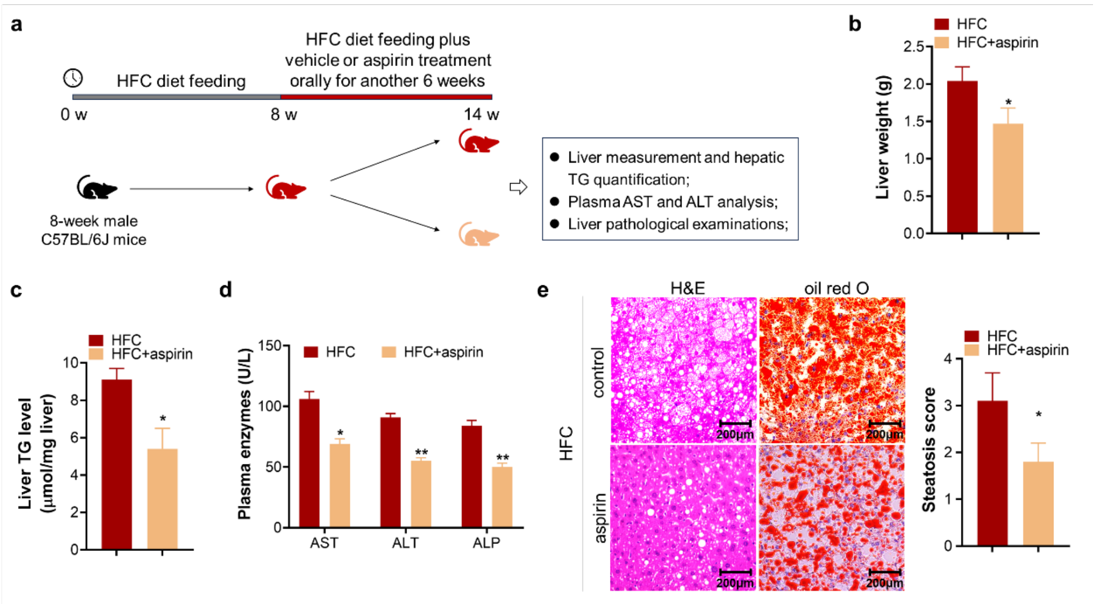

Supplementary Fig. 9. Evaluation of the anti-MASLD effects of antiplatelet agent aspirin in HFC diet-induced mice. Male C57BL/6J mice (8- week-old) were fed with HFC diet for 14 weeks in the presence of saline or aspirin (1.5 mg/kg BW) treatment for last 6 weeks, livers and plasma were collected for indicated analysis. (a) Schematic diagram of aspirin treatment in mice. (b) Liver weight measurement. (c) Quantification of hepatic TG content. (d) Measurement of plasma AST, ALT, and ALT levels. (e) H&E and oil-red O staining in liver. Scale bar, 200 µm. n = 5 mice per group. * $p < 0.05$, ** $p < 0.01$, compared with HFC diet-fed control mice.

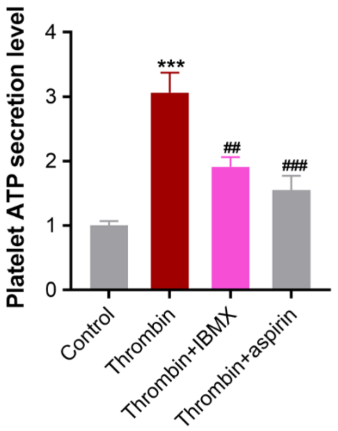

Supplementary Fig. 10. Pan PDE inhibitor IBMX inhibits ATP releasing in thrombin stimulated platelets. N = 4 independent biological experiments. * $p < 0.05$, ** $p < 0.01$, *** $p < 0.001$, *vs* the control group; ## $p < 0.01$, ### $p < 0.001$, *vs* the thrombin treated group.

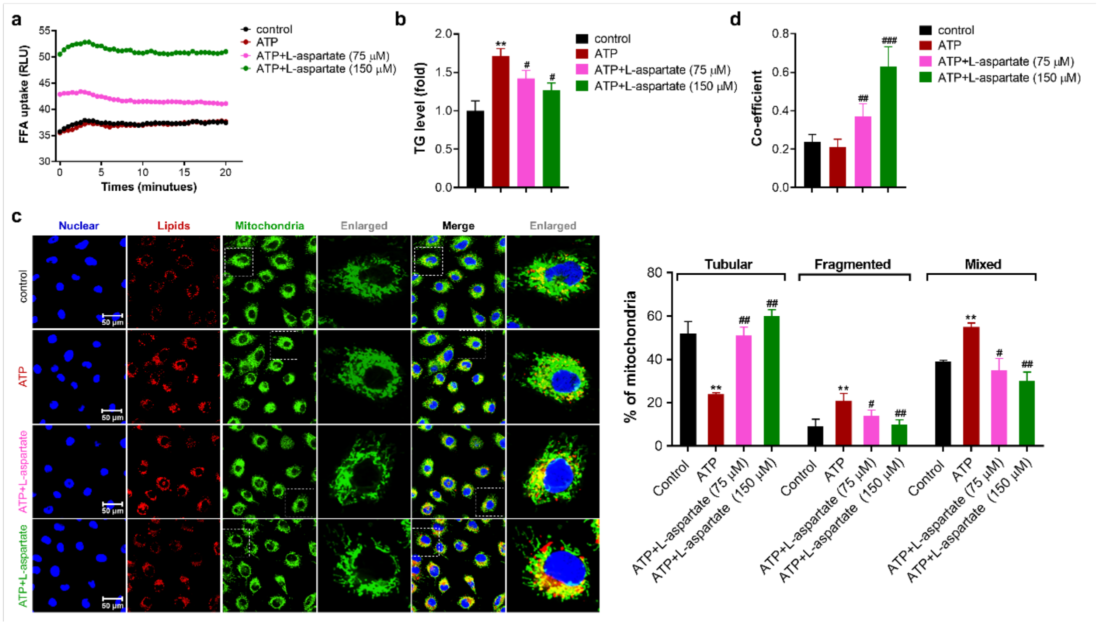

Supplementary Fig. 11. L-aspartate inhibits ATP induced lipid accumulation and mitochondrial fragmentation. (a) FFA uptake assay. (b) Cellular TG quantification. (c) Imaging lipids and mitochondria in hepatocytes, and mitochondrial morphology quantification. (d) Co-localization efficient analysis. N = 4 independent biological experiments. * $p < 0.05$, ** $p < 0.01$, *** $p < 0.001$, *vs* control cells; $^{\#}$ $p < 0.05$, $^{\#\#}$ $p < 0.01$, $^{\#\#\#}$ $p < 0.001$, *vs* OA-treated hepatocytes.

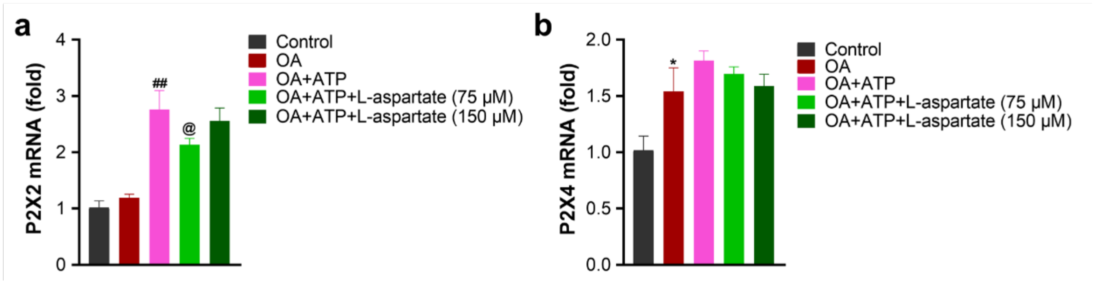

Supplementary Fig. 12. Examination of the effect of L-aspartate on the mRNA level of ATP receptors in hepatocytes upon OA or OA+ATP-treatment. (a) P2X2 mRNA level. (b) P2X4 mRNA level. * $p < 0.05$, *vs* control cells; # $p < 0.05$, ## $p < 0.01$, *vs* OA-treated hepatocytes; @ $p < 0.05$, *vs* OA+ATP-treated hepatocytes.

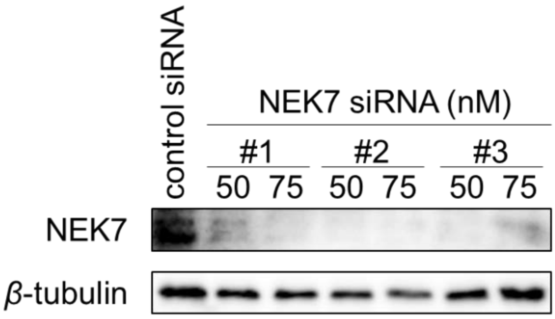

Supplementary Fig. 13. Examination of the efficacy of NEK7 knockdown in hepatocytes. Hepatocytes were transfected with control siRNA or NEK7 siRNA (50 or 75 nM) for 24 h, then cells were harvested for examination of NEK7 protein level by western blot.

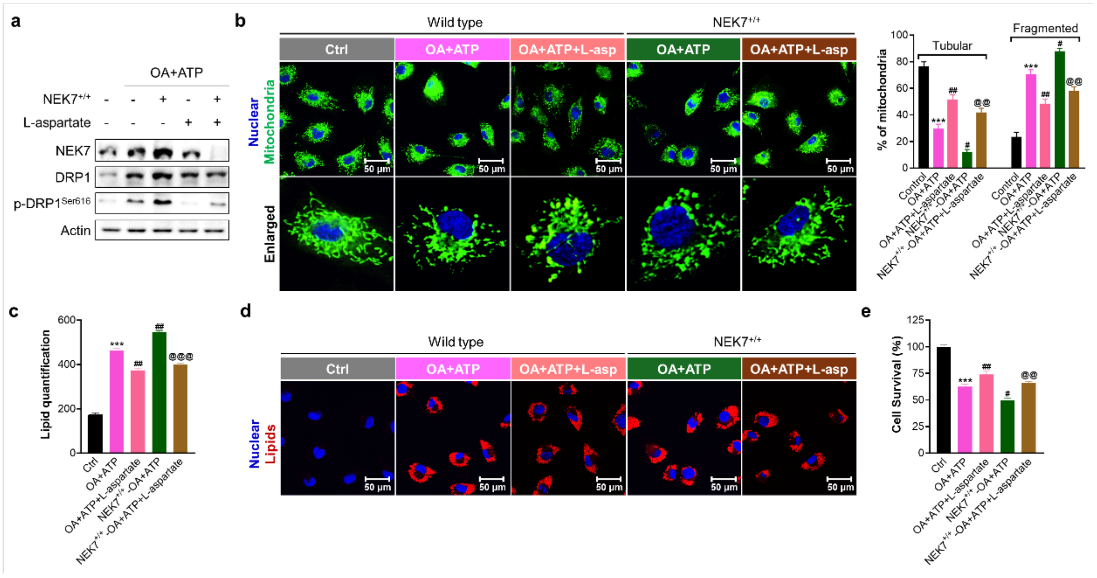

Supplementary Fig. 14. L-aspartate reversed mitochondrial fragmentation and lipid accumulation in NEK7$^{+/+}$-hepatocytes upon OA+ATP treatment. (a) Expression of NEK1/DRP1 axis related proteins. (b) Imaging mitochondria in hepatocytes and quantification. Scale bar, 50 µm. (c-d) Cellular TG level quantification and imagination. (e) Cell survival measurement. * $p < 0.05$, ** $p < 0.01$, *** $p < 0.001$, *vs* control cells; $^{\#}$ $p < 0.05$, $^{\#\#}$ $p < 0.01$, $^{\#\#\#}$ $p < 0.001$, *vs* OA+ATP-treated hepatocytes; $^{@}$ $p < 0.05$, $^{@@}$ $p < 0.05$, $^{@@@}$ $p < 0.05$, *vs* OA+ATP-treated NEK7$^{+/+}$-hepatocytes.

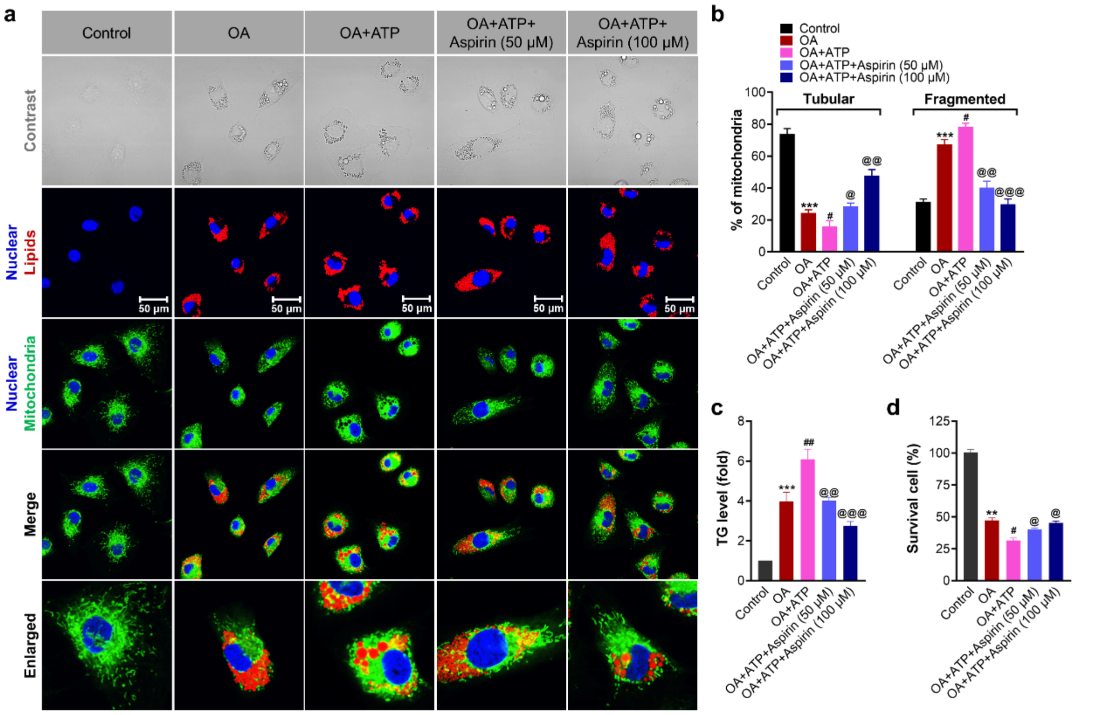

Supplementary Fig. 15. Determination of the effect of aspirin in mitochondrial fragmentation and lipid accumulation in OA+ATP-treated hepatocytes. (a-b) Imaging cellular lipids and mitochondria in hepatocytes, and quantification. Scale bar, 50 μm. (c-d) Cellular TG level and cell survival measurement. * $p < 0.05$, ** $p < 0.01$, *** $p < 0.001$, *vs* control cells; $^{\#} p < 0.05$, $^{\#\#} p < 0.01$, $^{\#\#\#} p < 0.001$, *vs* OA-treated hepatocytes; $^{@} p < 0.05$, $^{@@} p < 0.05$, $^{@@@} p < 0.05$, *vs* OA+ATP-treated hepatocytes.

Supplementary Table 1. The used anti-body information

Antibody	Company	Catalog
NIPSNAP	Affinity	Cat#DF15696
BNIP3	Proteintech,	Cat#68091-1
PINK1	Affinity	Cat# DF7742
Parkin	Bioss	Cat#bs-23687R
OPA1	Abcam	Cat# Ab157457
LC3	Abcam	Cat#ab192890
TOMM20	Affinity	Cat#DF4179
TFAM	Bioss	Cat#bs-8520R
PGC-1α	Santa Cruz Biotechnology	Cat#sc-518025
pLKB1^{Ser431}	Santa Cruz Biotechnology	Cat#sc-271924
pAMPKα^{Thr172}	Affinity	Cat#AF3423
CPT-1β	Bioss	Cat#bs-5045R
CHOP	Affinity	Cat#DF6025
NEK7	Affinity	Cat#DF4467
DRP1	Abcam	Cat#Ab314755
pDRP1^{Ser616}	Abcam	Cat# Ab314755
CD68	Abcam	Cat#Ab125212
Collagen I	Affinity	Cat#7001
CD42b	Abcam	Cat#Ab183345
β-Actin	Affinity	Cat#AF7018

Supplementary Table 2. The fomulation of the high fat diet and high cholesterol (HFC) diet

Product #	M21051101	
	gm%	kcal%
Protein	26.2	19.85
Carbonhydrate	26.3	19.85
Fat	34.9	60
Cholesterol	0.095	0.3
Total kcal/gm		100
Ingredient	gm	kcal
Casein, 80 Mesh	200	800
L-cystine	3	12
Com starch	0	0
Maltodextrin 10	125	500
Sucrose	68.8	275.2
Fructose	0	0
Cellulose, BW200	50	0
Soybeam Oil	25	225
Lard*	245	2205
Mineral Mix, S10026	10	0
DiCalcium Phosphate	13	0
Calcium Carbonate	5.5	0
Potassium Citrate, 1 H_2O	16.5	0
Vitamin Mix, V10001	10	40
Choline Bitartrate	2	0
FD＆C Blue Dye #1	0.05	0
Total	773.85	4057

Formulated by E. A. Ulman, Ph.D., Research Diets, Inc., 8/26/98 and 3/11/99.

*Typical analysis of cholesterol in lard = 0.95 mg/gram.

Cholesterol (mg)/4057 kcal = 232.8

Cholesterol (mg)/kg = 300.8

Supplementary Table 3. Significant metabolites in L-aspartate treated MAFLD mice (MASLD vs MASLD+L-aspartate)

Compounds	VIP	Fold change	Type
Urobilin	1.99	4.39	up
7,12-diketocholic acid	1.48	2.92	up
Alpha-Mercholic Acid	1.08	2.32	up
2-hydroxyphenylacetic acid	1.04	2.04	up
4-Hydroxy-3-methylbenzoic acid	2.04	2.02	up
cGMP	1.52	0.5	down
Stearidonic Acid	1.72	0.49	down
2-(4-Hydroxyphenyl) ethanol	1.33	0.49	down
6-Hydroxynicotinic Acid	1.05	0.47	down
9,10-DiHOME	1.87	0.46	down
12,13-DiHOME	1.87	0.46	down
Urocanic Acid	1.15	0.43	down
Cis-Aconitic Acid	1.29	0.4	down
2,4-diacetamino-2,4,6-triphenoxy-D-mannopyranose	1.54	0.38	down
cAMP	1.07	0.34	down
1-Single Palm Essence	1.97	0.30	down
Arachidyl glycine	1.18	0.21	down

Fig. 3k

BINP3

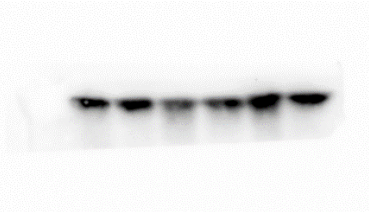

PINK1

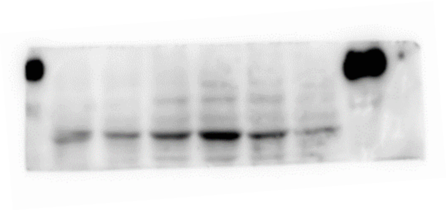

LC3

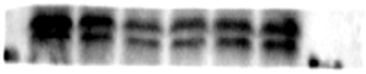

TFAM

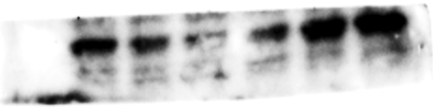

PGC-1α

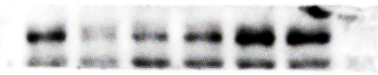

pLKB1^{Ser431}

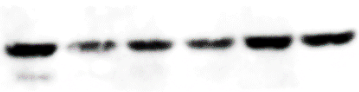

pAMPKα^{Thr172}

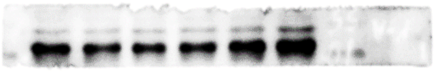

CPT-1β

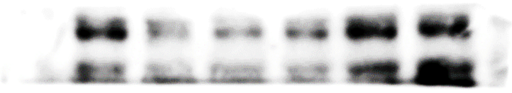

GAPDH

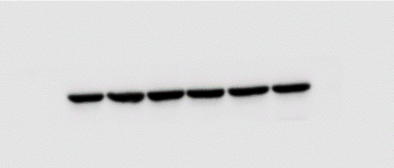

Fig. 5g

DRP1

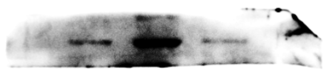

p-DRP1^{Ser616}

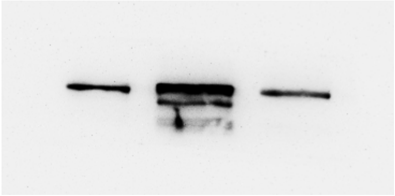

GAPDH

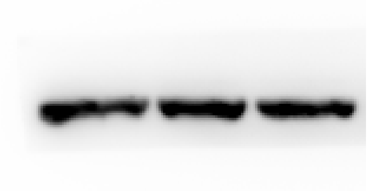

Fig. 6o

OPA1

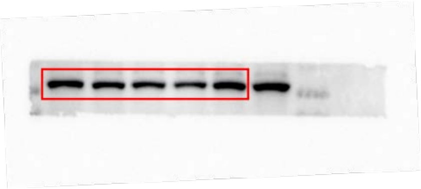

DRP1

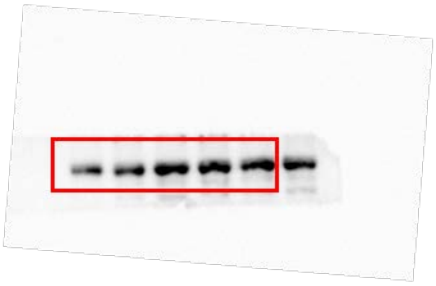

p-DRP1^{Ser616}

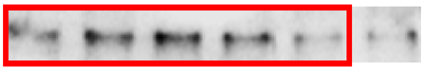

TOMM20

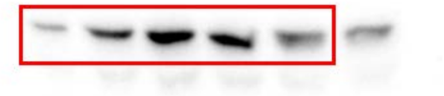

CHOP

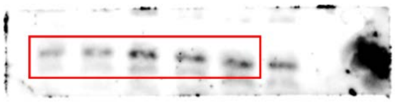

Actin

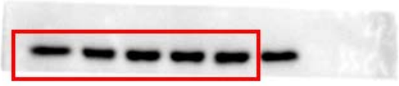

Fig. 6p

DRP1

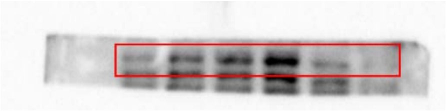

p-DRP1^{Ser616}

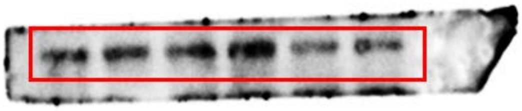

GAPDH

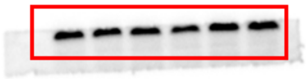

Fig. 7c

NEK7

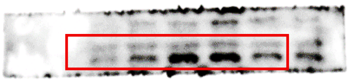

Actin

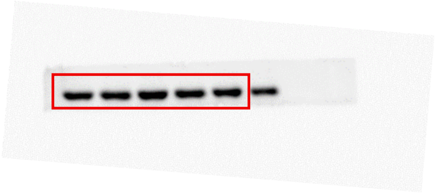

Fig. 7d

NEK7

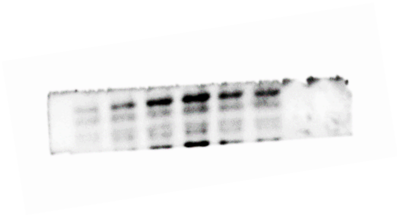

Actin

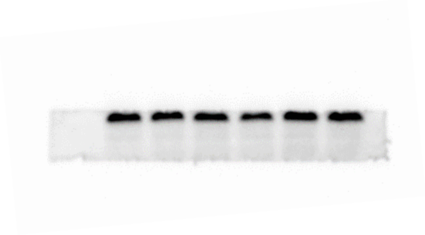

Fig. 7J

NEK7

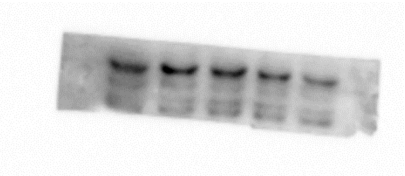

DRP1

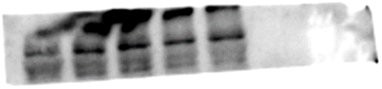

p-DRP1^{Ser616}

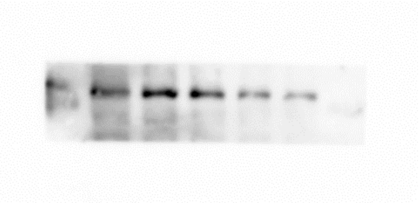

Actin

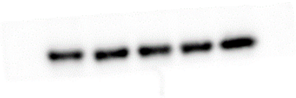

Fig. 7k

NEK7

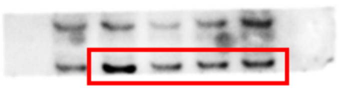

DRP1

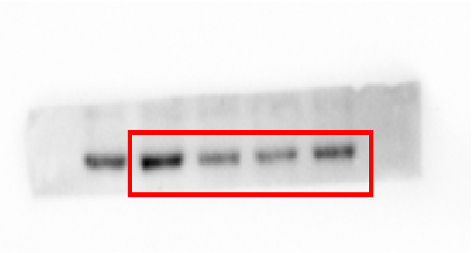

p-DRP1^{Ser616}

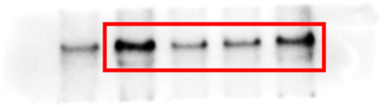

Actin

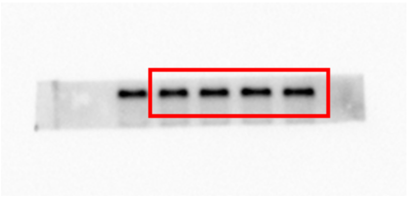

Supplement: Supplementary file 1 — Supplementary Information [file 12276_2026_1648_MOESM1_ESM.pdf]
